# Congenital Zika Syndrome Is Associated With Interferon Alfa Receptor 1

**DOI:** 10.3389/fimmu.2021.764746

**Published:** 2021-11-25

**Authors:** Tamiris Azamor, Daniela Prado Cunha, Andréa Marques Vieira da Silva, Ohanna Cavalcanti de Lima Bezerra, Marcelo Ribeiro-Alves, Thyago Leal Calvo, Fernanda de Souza Gomes Kehdy, Fernanda Saloum de Neves Manta, Thiago Gomes de Toledo Pinto, Laís Pereira Ferreira, Elyzabeth Avvad Portari, Letícia da Cunha Guida, Leonardo Gomes, Maria Elisabeth Lopes Moreira, Elizeu Fagundes de Carvalho, Cynthia Chester Cardoso, Marcelo Muller, Ana Paula Dinis Ano Bom, Patrícia Cristina da Costa Neves, Zilton Vasconcelos, Milton Ozório Moraes

**Affiliations:** ^1^ Laboratório de Hanseníase, Instituto Oswaldo Cruz, Fiocruz, Rio de Janeiro, Brazil; ^2^ Vice-Diretoria de Desenvolvimento Tecnológico, Instituto de Tecnologia em Imunobiológicos, Fiocruz, Rio de Janeiro, Brazil; ^3^ Unidade de Pesquisa Clínica, Instituto Nacional de Saúde da Mulher, da Criança e do Adolescente Fernandes Figueira, Fiocruz, Rio de Janeiro, Brazil; ^4^ Laboratório de Pesquisa Clínica em DST/AIDS, Instituto Nacional de Infectologia, Fiocruz, Rio de Janeiro, Brazil; ^5^ Laboratório de Diagnóstico por DNA, Universidade do Estado do Rio de Janeiro, Rio de Janeiro, Brazil; ^6^ Laboratório de Virologia Molecular, Universidade Federal do Rio de Janeiro, Rio de Janeiro, Brazil

**Keywords:** Congenital Zika Syndrome, rs2257167, placenta, type I interferon, type III interferon

## Abstract

Host factors that influence Congenital Zika Syndrome (CZS) outcome remain elusive. Interferons have been reported as the main antiviral factor in Zika and other flavivirus infections. Here, we accessed samples from 153 pregnant women (77 without and 76 with CZS) and 143 newborns (77 without and 66 with CZS) exposed to ZIKV conducted a case-control study to verify whether interferon alfa receptor 1 (*IFNAR1*) and interferon lambda 2 and 4 (*IFNL2/4*) single nucleotide polymorphisms (SNPs) contribute to CZS outcome, and characterized placenta gene expression profile at term. Newborns carrying CG/CC genotypes of rs2257167 in *IFNAR1* presented higher risk of developing CZS (OR=3.41; IC=1.35-8.60; *Pcorrected*=0.032). No association between *IFNL* SNPs and CZS was observed. Placenta from CZS cases displayed lower levels of *IFNL2* and *ISG15* along with higher *IFIT5.* The rs2257167 CG/CC placentas also demonstrated high levels of *IFIT5* and inflammation-related genes. We found CZS to be related with exacerbated type I IFN and insufficient type III IFN in placenta at term, forming an unbalanced response modulated by the *IFNAR1* rs2257167 genotype. Despite of the low sample size se findings shed light on the host-pathogen interaction focusing on the genetically regulated type I/type III IFN axis that could lead to better management of Zika and other TORCH (Toxoplasma, Others, Rubella, Cytomegalovirus, Herpes) congenital infections.

## Introduction

Zika virus (ZIKV) is a single stranded positive-sense RNA virus that belongs to the Flaviviridae family. Zika infection is mostly asymptomatic or associated with mild symptoms. After the outbreak in the Americas in 2015, the virus spread across 59 countries and more than 500.000 suspected cases were reported (1, 2). In a short while, there was a rise in cases of congenital abnormalities, including microcephaly, cerebral anomalies, congenital contractures, ocular alterations among other neurological abnormalities known as Congenital Zika Syndrome (CZS) (3–8). In a prospective cohort study, our group observed that 46% of the infants that were born to ZIKV-infected mothers bore abnormal clinical or brain imaging findings, including four infants with microcephaly, regardless of the trimester in pregnancy (7). Indeed, in a little while, a case-control study confirmed the association between the infection and CZS, and ZIKV epidemic was declared a public health emergence of international concern (3, 9). Nevertheless, not all infants that are born to ZIKV-infected mothers will develop CZS, and it is not clear what maternal and/or fetal factors contribute to infant adverse neurologic outcomes. One important risk factor for CSZ is infection within the first trimester of pregnancy, which poses almost twice as high a risk of severe outcomes such as CNS abnormalities when compared with third trimester infections (7). Furthermore, maternal nutritional and social factors, such as consumption of improper water and poor protein diet, have been related to CZS development (10, 11). These environmental factors do not completely explain CZS outcomes, and it has been reported that genetic background can influence these outcomes. Thus, studies testing other populations in Brazil identified maternal adenylate cyclase, and newborn collagen-encoding genes associated with CZS (12–14). Further, a previous study with a similar approach testing candidate SNPs demonstrated Toll-like receptor 3 (*TLR3*) rs3775291 and Tumor Necrosis Factor (*TNF*) rs1799964 associated with abnormal outcomes due to ZIKV infection during pregnancy (15).

During other congenital infections, namely TORCH (Toxoplasma, Others, Rubella, Cytomegalovirus, Herpes), which may cause congenital anomalies, placenta has been described as playing a crucial role in mother to fetus transmission (16). In zika, one of the hypotheses for the emergence of adverse neurological outcomes is that ZIKV can infect and cross trophoblast cell layers as cargo, ultimately reaching the fetal neurologic system and causing direct damage. On the other hand, ZIKV infection causes an innate immunological imbalance, excessive inflammation and vascular permeability dysfunction in the placenta, which may contribute to disrupting embryonic brain development (17–24).

Interferons (IFN) are key players of the innate immune response against viral infection, inducing hundreds of interferon-stimulated genes (ISGs) that act directly against virus components (25). Among these ISGs, ubiquitin-like protein ISG15, induced by type I IFNs, is one of the most strongly and rapidly induced, inhibiting viral replication and modulating host immunity (26–28). Another ISG, IFN-induced protein with tetratricopeptide repeats 5, IFIT5 (ISG58), activates IRF3/NF-κB pathway, which induces higher type I IFNs and proinflammatory mediators (29). It has been described that ZIKV disrupts type I IFN, harming phosphorylation of STAT1 and STAT2 (22, 30). In addition to a major role in antiviral defense, an exacerbated type I IFN response was demonstrated to be threatening for newborn development (31), indicating that a balanced production of type I IFNs could be effective in controlling infection and inflammation. Type III interferons (a.k.a. IFN-λ 1-4) present augmented expression during ZIKV infection in susceptible placental cells and higher levels of IFN-λ antagonize type I IFNs (21, 32–34). In this regard, administration of exogenous IFN-λ in mice led to signatures with balanced expression of ISGs (*IFI44L*, *OASL*, *OAS1*, and *MX1*) and inhibition of ZIKV replication, suggesting a therapeutic potential (35, 36).SNPs in the vicinity of *IFNAR1* and *IFNL1-4* loci have been associated with outcomes of viral infections, such as hepatitis B and C (37–39). Variants within *IFNAR1* have been associated with an error of innate immunity related to severe viscerotropic adverse events following vaccination with another flavivirus: attenuated yellow fever virus (40). The *IFNL4* rs12979860 CC genotype has been associated with persistent low levels of ISGs *IFIT1*, *IFIT2*, *IFIT3*, and *OAS1* in postpartum normal pregnancy (41). In another flavivirus infection, hepatitis C, *IFNL* SNPs rs12979860, rs8099917, rs8109886, and rs368234815 are markers for good prognosis in chronic patients treated with IFNα and ribavirin (39, 42–44).

In this paper, we describe the association between the genetic background of newborns and mothers from ZIKV-infected pregnancy and CZS development, focusing on SNPs in *IFNAR1*, *IFNL2* and *IFNL4* loci, as well as the functional consequences of specific genotypes for the immunological imbalance in at term placentas from pregnant women exposed to ZIKV.

## Materials and Methods

### Human Subjects and Sample Collection

Our studies made use of the ongoing prospective clinical cohort study of ZIKV+ pregnant women and their infants at maternal and child hospital (IFF/Fiocruz) in Rio de Janeiro, Brazil (IRB/CAAE: 52675616.0.000.5269). In this cohort, pregnant women who were ZIKV+ received their prenatal care at IFF/Fiocruz. Since December 2015, a total of 301 mothers who were suspected of having been infected by ZIKV during gestation were referred to IFF — a major public reference hospital in Rio de Janeiro for congenital infections and congenital anomalies. Here, we utilized a subpopulation of 143 newborns and 153 mothers from the IFF cohort, including 3 pairs of bi-chorionic and bi-amniotic twins, with availability of samples, and confirmed ZIKV infection during pregnancy by ZIKV PCR of urine, blood, or placenta samples from mothers or newborns as inclusion criteriaFrom those cases, 84 placentas (74 from congenital ZIKV infections and 10 from uninfected patients) were accessed, processed and analyzed for gene expression. Samples from mothers were tested for HIV, evidence of past Dengue virus (DENV) infection (by DENV IgG and IgM), and Chikungunya virus (CHIKV) (blood PCR). Maternal demographic, medical/prenatal history and clinical findings were entered into case-report forms. All infants underwent routine clinical and extensive neurologic evaluation at the time of birth and were tested for CHIKV infection (blood PCR), syphilis and TORCH infections (toxoplasmosis, rubella, CMV, and herpes simplex virus as determined by standard testing). Infants were evaluated for the following adverse neurologic outcomes: (a) microcephaly (head-circumference z score of less than -2), (b) abnormal brain imaging by pre- or post-natal ultrasound (e.g., computed tomography and/or magnetic resonance imaging), and/or (c) abnormal clinical examination (including neurologic, ocular, and/or auditory with abnormalities confirmed by a multidisciplinary team of neonatologists, neurologists, infectious disease specialists, geneticists, ophthalmologists, and physical therapists). Our study included ZIKV+ pregnant adult women >18 years of age and their infants. Exclusion criteria included maternal HIV infection and pregnancies complicated by other congenital infections, known to cause infant neurologic damage (e.g., TORCH, CHIKV). Placental samples were collected at the time of delivery from the umbilical cord insertion region and stored in RNA later until RNA extraction. For DNA analysis, 5 mL of blood was collected from pregnant women at study enrollment and an oral swab was collected from newborns. Rational and workflow of sample analysis in **Supplementary Figure 1**.

### Genetic Studies: SNP Selection and Linkage Disequilibrium Analysis

Selection of candidate SNPs for the case-control association study was performed by integrating different tools: Principal Component Analysis (PCA), ANNOVAR (45), allele frequencies, literature and HAPLOVIEW (46). First, all SNPs located in the *IFNL* (chr19:39,733,272-39,736,609-GRCh37/hg19) and *IFNAR1* regions (chr21:34,696,734-34,732,168- GRCh37/hg19) were recovered from African (ENS, GWD, LWK, MSL, and YRI) and European (CEU, FIN, GBR, IBS, and TSI) populations from phase 3 of the 1000 Genomes Project (47). Then, Principal Component Analysis (PCA) was performed using EIGENSOFT4.2 (46). The use of this strategy in the selection of functional SNPs assumes that, since the analyzed variability is of a functional genome region (meaning: a gene), the clusters generated by PCA would be mainly influenced by functionality. Thus, SNPs with high weight for principal component 1 (PC1) could be potential candidates for having a functional role. SNPs were thus sorted by decreasing values of “SNP weight” for PC1, and functional annotation of all SNPs was performed using ANNOVAR (45), with ref Gene hg19 (11 Dez 2015). According to the functional category identified by ANNOVAR, “SNP weight” for PC1 (with SNP weight values within the highest 30, called top SNPs), minimal allele frequencies (MAF) in African and European populations (> 0.1) and associations with infectious diseases already reported in the literature, SNPs present in the *IFNAR1* and *IFNL* region were selected for genotyping and haplotype construction. Haplotype inferences using selected SNPs, haplotype frequencies and linkage disequilibrium (LD) analysis for all studied populations were performed using HAPLOVIEW (46). To select SNPs, PCA was used to retrieve those located either in the *IFNAR1* or *IFNL* regions, which are found among the African and European populations from the 1000 Genomes Project (**Supplementary Figure 2A**). Within *IFNL*, we selected four representative SNPs (rs12979860, rs4803222, rs8109886, rs8099917, and rs368234815). In the *IFNAR1* region, SNPs rs2843710, rs2257167, rs17875834, rs2834202 were selected to construct the haplotypes in parental populations. Allele frequencies, annotation, and reference of the selected SNPs are described in **Supplementary Table 1**. Linkage disequilibrium (LD) analysis and haplotype arrangements indicated ancestry-specific patterns for these two genomic regions. (**Supplementary Figure 2B**). The *IFNAR1* arranged haplotypes suggest rs2843710, rs2257167 are tags to discriminate Europeans and Africans, while *IFNL* rs12979860 and rs8109886 SNPs also present very different frequencies among the major Brazilian parental populations (**Supplementary Table 2**).

### Genomic DNA Extraction and SNP Genotyping Analysis

DNA extraction was performed from saliva swabs or whole blood cells collected from each individual newborn (n=143) and mothers (n=153), respectively, using the salting out method. Following extraction, DNA was quantified with a Nanodrop ND 1000 spectrophotometer (Nanodrop Technologies). After PCA, LD and haplotype analysis, the following tag polymorphisms were genotyped due to their representativeness within the corresponding genomic regions: *IFNL*2-*IFNL*4: rs8099917 (C11710096_10) located 8.9 kb upstream of the *IFNL*4 (T > G) start codon and rs8109886 (C11710100_10) located 3.3 kb upstream of the *IFNL*4 (A > C) start codon; *IFNL*4: rs12979860 (C7820464_10) in intron 4 (C > T), rs4803222 (C7820457_10) in the 5’ UTR (C > G), and rs368234815 an indel in exon 1 (TT>ΔG) as described by Prokunina-Olsson *et al*, 2013 (44); *IFNAR1*: rs2257167 (C:16076297_10) located within exon 4 of *IFNAR1* (G>C) and rs2843710 (C:26796048_10) located within the promoter region of *IFNAR1* (C > G). All SNPs were genotyped using the allelic discrimination method for real-time TaqMan assays (Applied Biosystems) using Viia7 Real-time PCR System. Approximately 30 ng of DNA was used in the genotyping reaction. Statistical analyses were performed using “snpassoc”, “genetics” and “haplo.stats” packages in software R version 2.11.1, as previously described (48). Briefly, genotype frequencies were tested for HWE using a Chi-square test. The genotypic, allelic, and carrier frequencies were calculated and compared in cases and controls by conditional logistic regression adjusted for ancestry and trimester of infection. Next, we compared the frequencies between CZS and no CZS, separately. Linkage disequilibrium values for SNPs studied in *IFNAR1* and *IFNL* were estimated by r2 and haplotype frequencies were compared between cases and controls by logistic regression, also adjusted for ancestry and trimester of infection. For mother-child SNP interaction, we used Estimation of Maternal, Imprinting and Interaction Effects using multinomial modeling analysis using a multinomial model to test the existence (and estimate) of maternal genotype relative risk parameters that may increase (or decrease) the possibility that a child is affected, as described previously (49).

### Ancestry Analysis

Since the Brazilian population is highly admixed and ethnic classification is not uniformly defined, ancestry data is necessary to adjust the logistic regression and eliminate bias in genetic associations (50). Thus, DNA samples were genotyped for 46 Ancestry Informative Markers (AIM)-Indels in a multiplex PCR system followed by capillary electrophoresis in an ABI 3500 Genetic Analyzer (Thermo Fisher), as described previously (51, 52). Allele calls were obtained by GeneMapper v.4.1 and results for individual and global ancestry estimates were performed by using the HGDP-CEPH diversity panel as a reference (European, African and Native-American; K=3) in STRUCTURE v2.3. In the logistic regression performed in R, covariates AFR+EUR were used to control for population stratification along with trimester of infection.

### ZIKV PCR Detection

RT-qPCR was performed using the 2x QuantiTect Probe RT-PCR kit (Qiagen, Valencia, CA, USA) with the same primers and cycle times as previously described (53). All the assays were carried out in triplicate and fluorescence curves that crossed the threshold within or below 38 cycles were considered positive.

### Gene Expression Profile Analysis

Analysis of gene expression in placental tissue, specifically from the region of umbilical cord insertion, from pregnant mothers (control, with or without CZS samples) was performed using Fluidigm (Biomark platform) assays. Detailed data available under request. Our experimental design followed a previously described workflow (54).

### Real-Time RT-PCR Expression Analysis

From routines created in R for parsing raw foreground and background intensities, we carried out background correction and exploratory data analysis: fluorescence accumulation and melting curve graphs of Rn for each reaction with each gene. For relative quantification of expression, the fluorescence accumulation data of each sample were used for fitting four parameter sigmoid curves using the qPCRlibrary from R statistical package version 3.4.1 (48). For each amplification, the cycle of quantification was determined as the maximum of the second derivative of the fit sigmoid curve and the efficiency, as the ratio between the fluorescence of the cycle of quantification and the fluorescence of the cycle that immediately preceded that. For each gene, efficiency was estimated by the mean of all the efficiencies for each amplification reaction for that gene. Endogenous controls used for normalizing between different amplified samples were selected by the geNorm method. Normalization factors were estimated for each sample using the geometric average of the selected normalized genes (55).

### Statistical Analysis of Gene Expression

Pairwise comparisons of log-transformed (base 2) normalized expression means between/among groups of interest were performed by contrasts/differences (fold-changes) obtained after both bi- and multivariate linear models adjusted by ordinary least square regressions. Whenever the variable of interest had more than two levels, p-values were corrected by the Tukey Honest Significant Difference post-Hoc method (56). After gene-per-gene pairwise comparisons, we conducted a Type I error adjustment for multiple comparisons by the Holm-Bonferroni method (57). Different sets of confounding variables were selected by clinical experts and included in the multivariate models to adjust the fit effects for different variables of interest for all genes. To obtain the marginal means expected values for the variable of interest, we kept the confounding variables in the multivariate models in their mean values or equal proportions in all models. For the analysis, two-tailed levels of significance ≤ 0.01, 0.05, and 0.1 were considered as “highly significant”, “significant”, and “suggestive”, respectively.

## Results

### Newborn *IFNAR1* rs2257167 Are Associated With CZS Outcome

The DNA samples from whole blood admixed ancestry population of 143 newborns and 153 mothers from ZIKV-infected pregnancies, with development of CZS (cases) or otherwise (controls) (**Supplementary Tables 3, 4**), were genotyped for SNPs encompassing *IFNAR1* (rs2257167, rs2843710, rs2834202, and rs17875834), *IFNL4* genes (rs12979860, rs368234815, and rs4803222), and within *IFNL2* and *IFNL4* genes (rs8099917 and rs8109886). The frequency of each SNP was verified in cases and controls and CZS outcome was evaluated. Genotype frequencies were found to be in HWE for all SNPs tested. Data were adjusted by genetic ancestry and the trimester of pregnancy in which ZIKV infection occurred (when symptoms of ZIKV infection were detected). CZS risk was observed for CG/CC carriers of SNP rs2257167 following FDR correction, OR = 3.42; CI = 1.35-8.6, *Pcorrected* = 0.032 (**Table 1**). No significant differences were observed in the frequencies between cases and controls in any other SNP tested. Other analyses did not show any significant results including: *IFNAR1* newborn haplotypes, *IFNAR1* mother genotypes and haplotypes, *IFNL* genotypes or haplotypes, as well as the Estimation of Maternal, Imprinting and Interaction Effects using multinomial modeling analysis to investigate if risk-associated newborn genotypes could possibly present an additive contribution of maternal same genotype (**Supplementary Tables 5–7**).

**Table 1:** Association study with newborn *IFNAR1* and *IFNL* SNPs and CZS abnormalities.

	No CZS findings		CZS		Adjusted by trimester of ZIKV exposure and ancestry				
	N	%	N	%	OR	lower	upper	p-value	FDR p-value
**rs2257167**									
G/G	56	73.7	38	58.5	1			0.023	0.068
C/G	18	23.7	24	36.9	3.13	1.19	8.21		
C/C	2	2.6	3	4.6	5.72	0.77	42.47		
G/G	56	73.7	38	58.5	1			0.007	0.032
C/G-C/C	20	26.3	27	41.5	3.41	1.35	8.6		
G/G-C/G	74	97.4	62	95.4	1			0.171	0.308
C/C	2	2.6	3	4.6	3.83	0.55	26.97		
G/G-C/C	58	76.3	41	63.1	1			0.032	0.072
C/G	18	23.7	24	36.9	2.71	1.07	6.92		
Additive	76	53.9	65	46.1	2.76	1.29	5.91	0.006	0.032
									
**rs2843710**									
G/G	30	40	24	36.4	1			0.210	0.629
C/G	36	48	27	40.9	1.15	0.47	2.82		
C/C	9	12	15	22.7	2.65	0.85	8.31		
C/C	30	40	24	36.4	1			0.340	0.765
C/G-G/G	45	60	42	63.6	1.49	0.65	3.41		
C/C-C/G	66	88	51	77.3	1			0.082	0.525
G/G	9	12	15	22.7	2.46	0.87	6.92		
C/C-G/G	39	52	39	59.1	1			0.641	1.000
C/G	36	48	27	40.9	0.83	0.37	1.85		
Additive	75	53.2	66	46.8	1.55	0.89	2.69	0.117	0.525
**rs2834202**									
A/A	44	58.7	40	62.5	1			0.398	1
A/G	29	38.7	20	31.2	0.87	0.37	2.04		
G/G	2	2.7	4	6.2	3.26	0.48	21.89		
A/A	44	58.7	40	62.5	1			0.923	1
A/G-G/G	31	41.3	24	37.5	1.04	0.46	2.33		
A/A-A/G	73	97.3	60	93.8	1			0.186	1
G/G	2	2.7	4	6.2	3.41	0.52	22.42		
A/A-G/G	46	61.3	44	68.8	1			0.605	1
A/G	29	38.7	20	31.2	0.8	0.35	1.85		
Additive	75	54	64	46	1.21	0.62	2.36	0.567	1
**rs17875834**									
C/C	72	93.5	56	88.9	1			0.162	0.729
C/T	5	6.5	7	11.1	2.76	0.63	12.06		
Additive	77	55	63	45	2.76	0.63	12.06	0.162	0.729
**rs12979860**									
C/C	31	40.3	22	33.3	1			0.608	1
C/T	34	44.2	28	42.4	1.3	0.54	3.15		
T/T	12	15.6	16	24.2	1.78	0.56	5.68		
C/C	31	40.3	22	33.3	1			0.406	1
C/T-T/T	46	59.7	44	66.7	1.42	0.62	3.25		
C/C-C/T	65	84.4	50	75.8	1			0.419	1
T/T	12	15.6	16	24.2	1.54	0.54	4.38		
C/C-T/T	43	55.8	38	57.6	1			0.858	1
C/T	34	44.2	28	42.4	1.08	0.48	2.39		
Additive	77	53.8	66	46.2	1.33	0.76	2.33	0.319	1
**rs8099917**									
T/T	58	75.3	43	65.2	1			0.309	0.695
G/T	17	22.1	18	27.3	2.09	0.78	5.61		
G/G	2	2.6	5	7.6	1.66	0.27	10.11		
T/T	58	75.3	43	65.2	1			0.130	0.542
G/T-G/G	19	24.7	23	34.8	2	0.81	4.95		
T/T-G/T	75	97.4	61	92.4	1			0.706	1
G/G	2	2.6	5	7.6	1.41	0.23	8.44		
T/T-G/G	60	77.9	48	72.7	1			0.154	0.542
G/T	17	22.1	18	27.3	2.02	0.76	5.41		
Additive	77	53.8	66	46.2	1.63	0.78	3.37	0.181	0.542
**rs8109886**									
A/A	24	31.2	22	33.3	1			0.795	1
C/A	36	46.8	31	47	0.86	0.34	2.16		
C/C	17	22.1	13	19.7	0.68	0.22	2.11		
A/A	24	31.2	22	33.3	1			0.622	1
C/A-C/C	53	68.8	44	66.7	0.8	0.34	1.91		
A/A-C/A	60	77.9	53	80.3	1			0.548	1
C/C	17	22.1	13	19.7	0.74	0.28	1.98		
A/A-C/C	41	53.2	35	53	1			0.971	1
C/A	36	46.8	31	47	1.01	0.46	2.23		
Additive	77	53.8	66	46.2	0.83	0.47	1.45	0.505	1
**rs4803222**									
G/G	33	42.9	33	50.8	1			0.909	1
C/G	39	50.6	26	40	0.84	0.36	1.92		
C/C	5	6.5	6	9.2	0.84	0.2	3.56		
G/G	33	42.9	33	50.8	1			0.662	1
C/G-C/C	44	57.1	32	49.2	0.84	0.38	1.86		
G/G-C/G	72	93.5	59	90.8	1			0.904	1
C/C	5	6.5	6	9.2	0.92	0.23	3.67		
G/G-C/C	38	49.4	39	60	1			0.713	1
C/G	39	50.6	26	40	0.86	0.39	1.91		
Additive	77	54.2	65	45.8	0.88	0.48	1.64	0.694	1
**rs368234815**									
TT/TT	34	44.2	24	37.5	1			0.872	1
TT/ΔG	28	36.4	27	42.2	1.22	0.5	2.95		
ΔG/ΔG	15	19.5	13	20.3	1.27	0.43	3.72		
TT/TT	34	44.2	24	37.5	1			0.604	1
TT/ΔG-ΔG/ΔG	43	55.8	40	62.5	1.24	0.55	2.76		
TT/TT-TT/ΔG	62	80.5	51	79.7	1			0.775	1
ΔG/ΔG	15	19.5	13	20.3	1.16	0.43	3.1		
TT/TT-ΔG/ΔG	49	63.6	37	57.8	1			0.771	1
TT/ΔG	28	36.4	27	42.2	1.13	0.5	2.54		
Additive	77	54.6	64	45.4	1.14	0.68	1.92	0.624	1

DNA samples from 143 newborns with congenital infection by ZIKV, with or without CZS, were genotyped for IFNAR1 SNPs rs2257167, rs2843710, rs2834202, and rs17875834; and IFNL SNPs rs8109886, rs12979860 rs8099917, rs4803222, and rs368234815. Data were adjusted considering the trimester of the first symptoms or asymptomatic ZIKV infections and the percentage of the African and European ancestry of each individual. The total number of genotyped samples for each SNP may vary due to genotype miscalling. Major allele was used as baseline. Odds ratio (OR) with 95% confidence interval (CI) and p-values. We conducted type I error adjustment of multiple comparisons by the False Discovery Rate (FDR) method.

### *IFNAR1* rs2257167 CG/CC Genotypes as CZS Risk Factors in ZIKV Infection During Second and Third Trimester of Pregnancy

**Figure 1:**
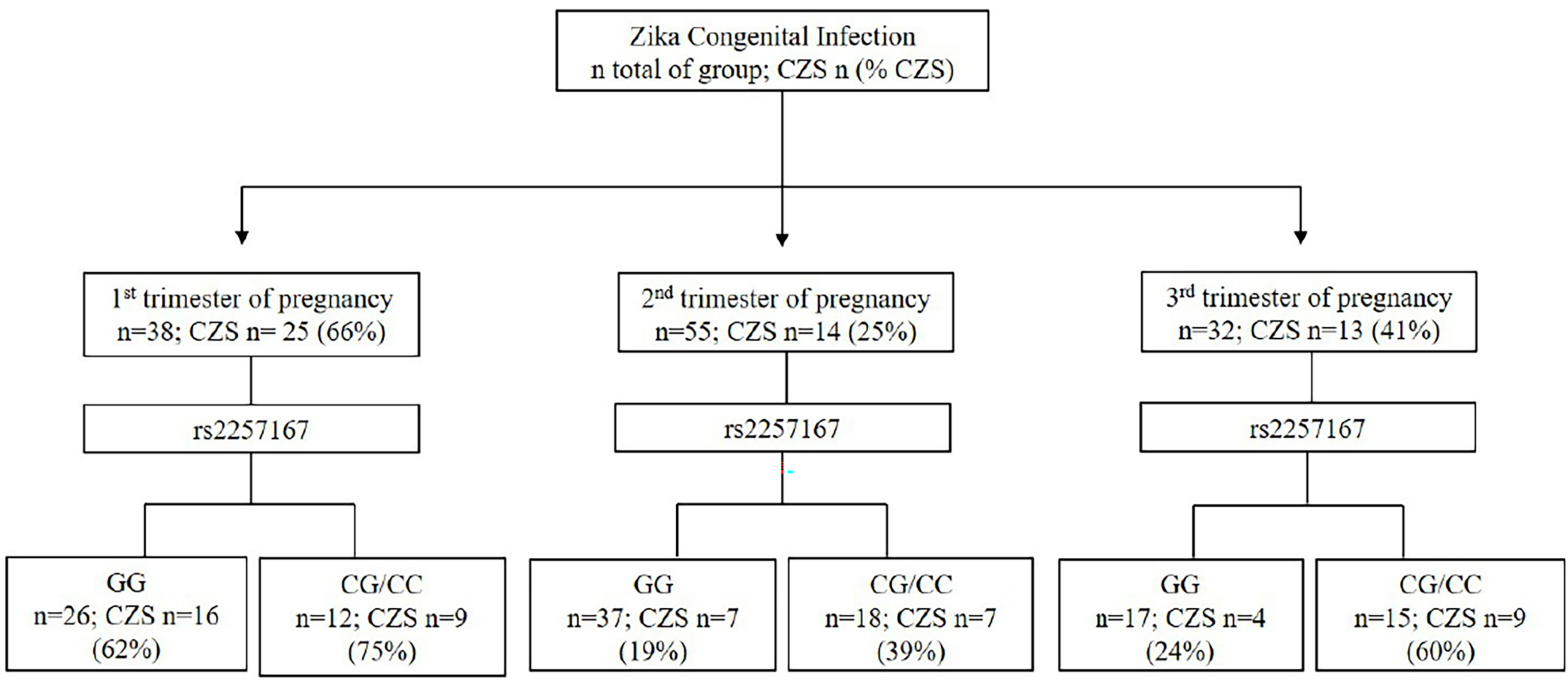
Event-based flowchart of CZS occurrence. The trimester of pregnancy in which first Zika symptoms occur and newborn genotypes of rs2257167 were used as independent variables to determine the association with CZS outcome. Total number of newborns with full information of both trimesters of infection and rs2257167 genotypes (n=125) was used to calculate the absolute number of newborns per group (n) and CZS percentage. Concerning infections in the first trimester of pregnancy, we found that irrespective of rs2257167 genotypes, 66% of the cases developed CZS, in contrast with second and third trimesters. Considering rs2257167, newborn CG/CC genotype seems to be associated with CZS risk in the second and third trimesters.

Following previous clinical studies (7), in our cohort, the determination of the trimester of pregnancy in which ZIKV infection occurs was a strong predicting factor for CZS outcome, along with *IFNAR1* genotype (**Figure 1**). Taking into account only newborns with information of rs2257167 genotype and trimester of infection (n=125), results show that ZIKV infection during first trimester of pregnancy culminates in with 66% of CZS cases, in contrast with second (25%) or third (41%) trimesters. Interestingly, data showed that newborns with rs2257167 CG/CC genotypes presented twice the frequency compared with newborns with the GG genotype in second (19%-GG *vs* 39% -CG/CC) and third (24%-GG *vs* 60%-CG/CC) trimesters.

### Congenital ZIKV Infection Leads to an Immunological Imbalance in Placenta

To functionally verify how ZIKV could influence severe congenital outcomes across associated *IFNAR1* genotypes, we performed gene expression analyses from the placental tissues, most of which were fetal, obtained at time of delivery from 10 uninfected pregnant woman and 74 congenital ZIKV cases (**Supplementary Table 8**). First, all ZIKV RT-PCR positive samples at term showed higher gene expression of most genes analyzed. ZIKV congenital infections occurring in the third trimester of pregnancy resulted in highest gene expression levels. Finally, mothers exhibiting ≥ 40 years of age (y/o) expressed lower levels of inflammatory genes(**Supplementary Table 9**). Because of these intrinsic differential expression profiles, ZIKV RT-qPCR positive placenta, trimester of exposure to ZIKV, and mothers’ age (≥ 40 y/o) were considered as variables in gene expression analysis of congenital ZIKV cases.

**Figure 2:**
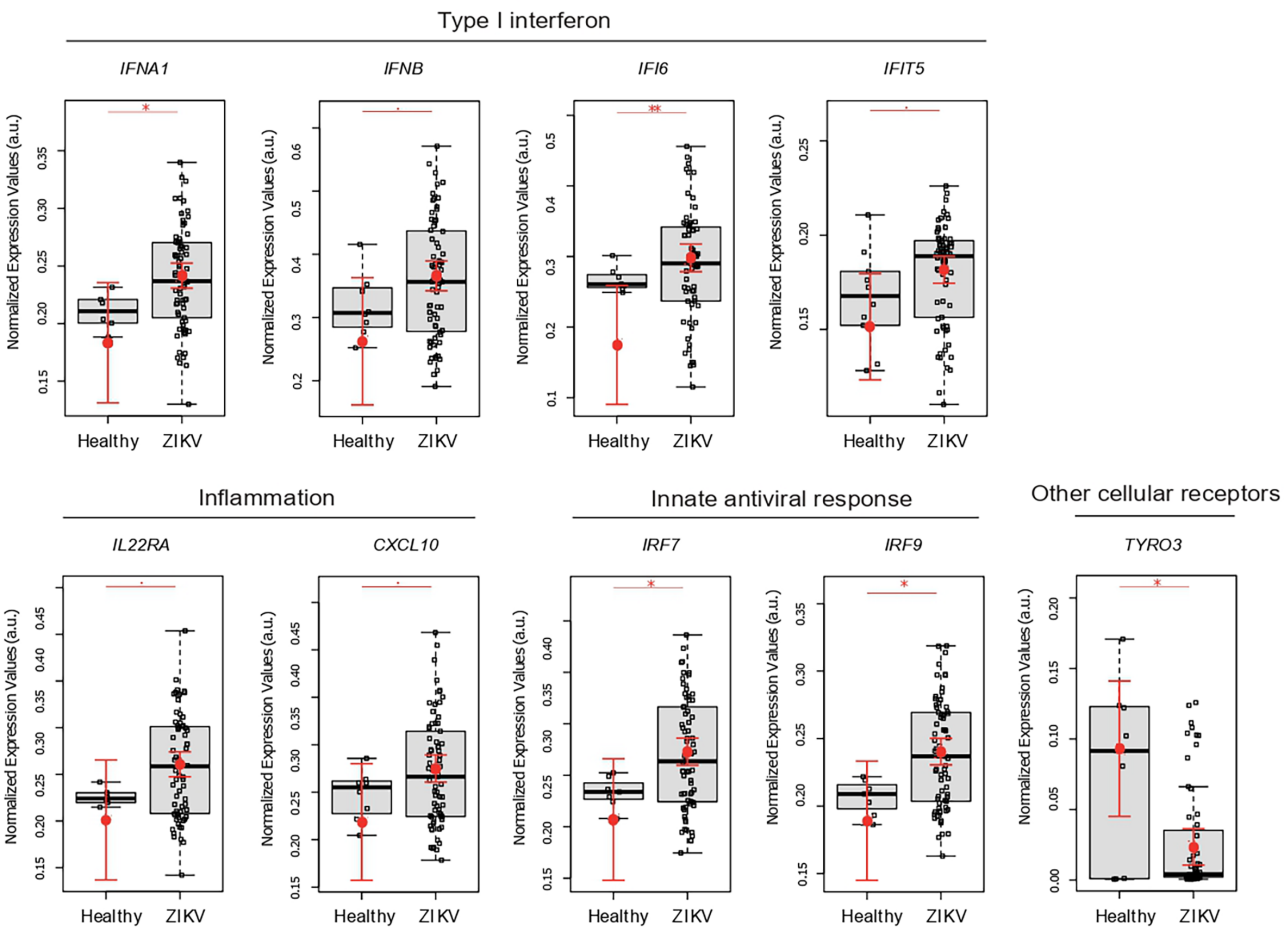
ZIKV infection leads to an immunological imbalance in placenta. Placental gene expression profile in healthy (N = 10) versus ZIKV-infected women (N = 74). Each dot corresponds to one placenta analyzed. The number of dots varies according to gene analyzed due to failed amplifications. Median and standard deviation of gene expression values are normalized by the housekeeping genes selected by the geNorm and NormFinder as well as *18S* ribosomal RNA and *RLP13* ribosomal protein L13 (grey boxes). Values are adjusted by mothers’ age (below or equal to/above 40 years of age) and trimester of infection (the trimester of pregnancy in which the first Zika symptoms occur or asymptomatic ZIKV infections). P-values ** ≤ 0.01, * ≤ 0.05 and ≤ 0.1.

By comparing placental gene expression from congenital ZIKV infections *vs* uninfected pregnant women, in spite of small sample number in this group, results showed that ZIKV leads to a typical inflammatory response in the placenta. This response includes higher expression of: anti-viral type I IFN genes (*IFIT5*, *IFNA1*, and *IFNB*), type II interferon (*IFI16*), cytokine signaling (*IL22RA* and *IP10*), and interferon regulatory factors (*IRF7* and *IRF9*); together with decreased expression of *TYRO3* (**Figure 2**).

### Decreased *IFNL2* and Augmented Type I IFN in Placenta Aa Term Is Associated With Newborn CZS Abnormalities

**Figure 3:**
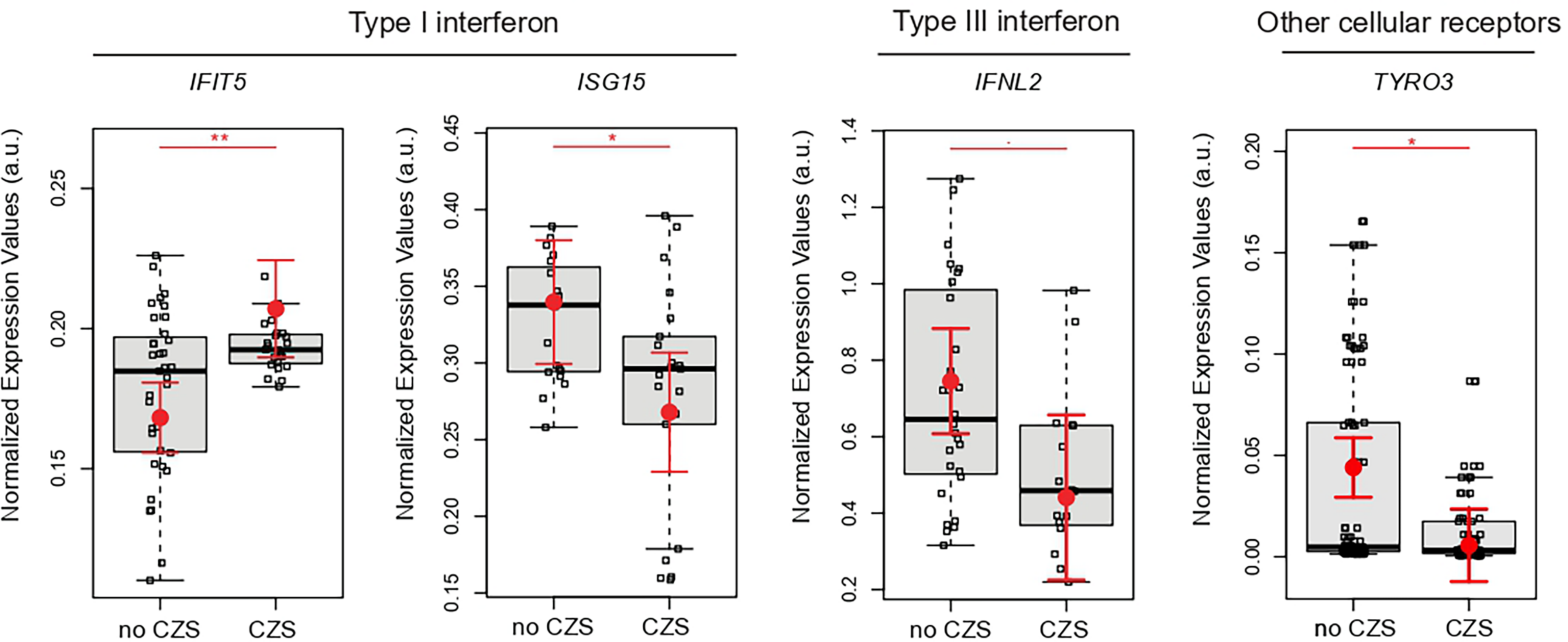
Placental gene expression associated with CZS. Detailed graphs of differentially expressed genes in placenta without CZS (No CZS; N = 45) or with CZS (CZS; N = 29). Each dot corresponds to one placenta analyzed. The number of dots varies according to gene analyzed due to failed amplifications. Median and standard deviation of gene expression values are normalized by housekeeping genes selected by geNorm and NormFinder as well as *18S* ribosomal RNA and *RLP13* ribosomal protein L13 (grey boxes). Values are adjusted by mothers’ age (below or equal to/above 40 years of age) and infection trimester (trimester of pregnancy in which the first Zika symptoms occur or asymptomatic ZIKV infections). P-values ** ≤ 0.01, * ≤ 0.05, and ≤0.1.

Next, we tested whether gene expression signatures of placentas from ZIKV-infected women could be associated with the presence or absence of CZS. These analyses illustrated *IFNL2*, *ISG15*, and *TYRO3* significant decreases in newborns with CZS. On the other hand, *IFIT5* increased significantly in newborns in the CZS group (**Figure 3**).

### Genotypes rs2257167 CG/CC Are Associated With Increased Placental Type I IFN and Inflammatory Response

**Figure 4:**
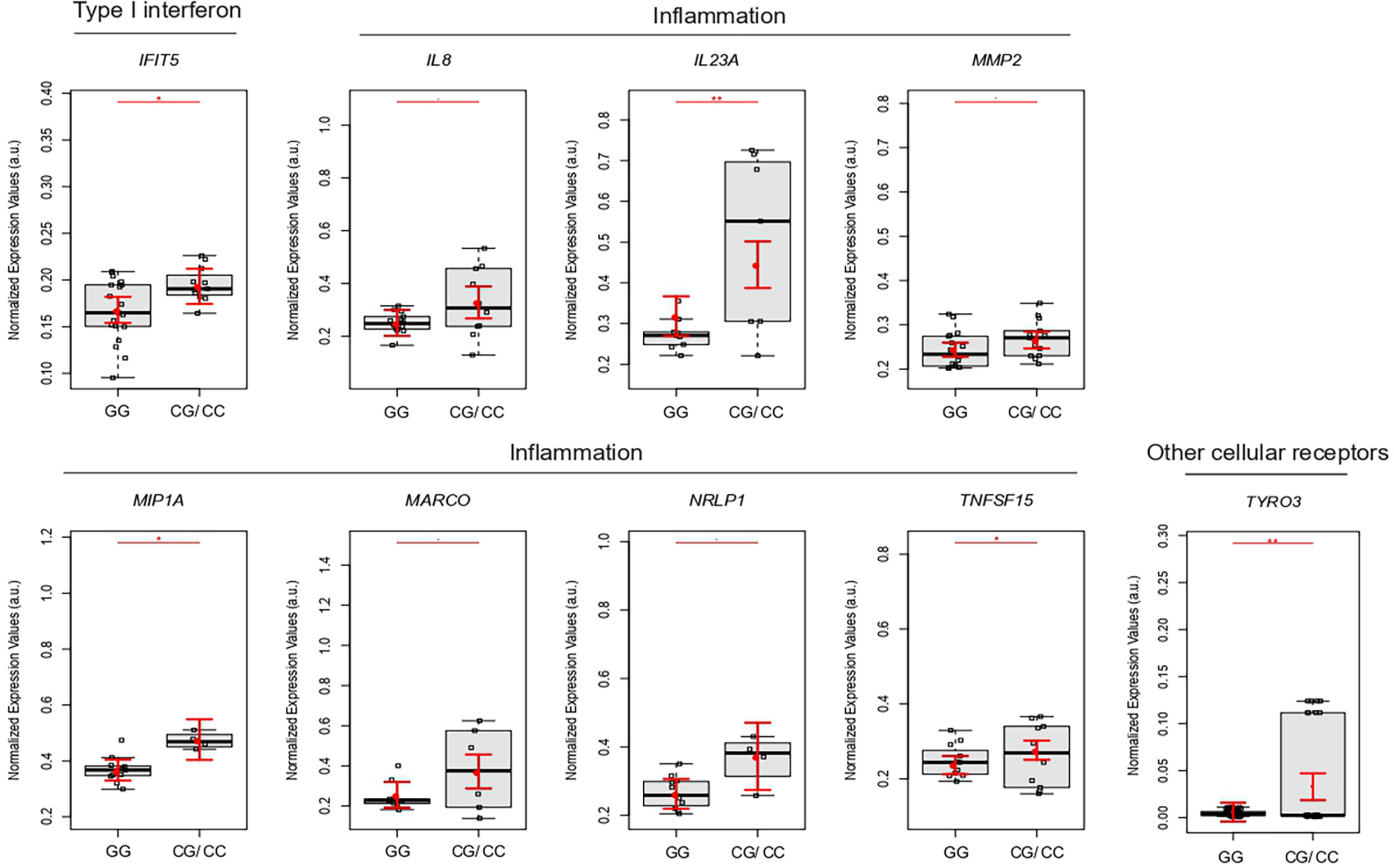
Placental gene expression is modulated by newborn rs2257167 genotypes in ZIKV-infected pregnancy. Detailed graphs of differentially expressed genes in placenta from rs2257167 GG (N = 20) and CG/CC (N = 13) newborns. Each dot corresponds to one placenta analyzed. The number of dots varies according to gene analyzed due to failed amplifications. Median and standard deviation of gene expression values are normalized by housekeeping genes selected by geNorm and NormFinder as well as *18S* ribosomal RNA and *RLP13* ribosomal protein L13 (grey boxes). Values are adjusted by mothers’ age (below or equal to/above 40 years of age) and infection trimester (trimester of pregnancy in which first Zika symptoms or asymptomatic ZIKV infections occur) P-values**≤ 0.01, * ≤ 0.05, and ≤ 0.1.

We clustered 39 newborns and 45 mothers according to GG or CG/CC genotypes of rs2257167 to assess how *IFNAR1* newborn background influences the placental gene expression profile. Placentas from rs2257167 CG/GG newborns showed significantly increased expression of *IFIT5* and genes related with the inflammatory response (*IL8, IL23A, MMP9, MIP1A, MARCO, NRLP1, and TNFSF15*) (**Figure 4**). We could highlight also that placentas from GC/CC non-CZS babies have higher levels of most of the genes statistically different when compared to i) GG non-CZS babies or ii) GC/GG CZS newborns. One important marker is *MIP1A* that is significantly augmented in GC/CC compared with GG newborns with CZS (**Supplementary Figure 3**)

## Discussion

Despite the high risk, ZIKV infection during pregnancy is necessary, albeit not enough, to induce CZS. We hypothesized whether host genetic background, especially SNPs in *IFNAR1* and *IFNL*, contributes to CSZ development and conducted a case-control study with CZS cases and healthy ZIKV+ mothers. Specifically, our results demonstrated that newborns who carried the CG/CC genotypes of SNP rs2257167 (*IFNAR1*) had a 3.4 higher risk of developing CZS, resulting from ZIKV infection during pregnancy, compared to those with the GG genotype. In chronic hepatitis B infected patients, the presence of the C allele was associated with higher plasma levels of the aspartate and alanine amino-transferase hepatic enzymes (58). Another study among chronic HBV-infected patients suggested that patients carrying the rs2257167 CC (leu/leu) genotype presented higher expression levels of *IFNAR1* in PBMCs when compared with patients carrying the GG genotype (val/val) (59). Moreover, crystal structure studies showed that Val141 is distal to the ligand binding surface, hence Val141 (G)> Leu141(C) may influence the *IFNAR1* downstream signaling (59). Here we observed that individuals Leu141(C) carriers presented a high *IFIT5* and inflammatory placenta profile.

Our cohort also highlights ZIKV infection in the first trimester of pregnancy as a critical CZS risk-associated factor, and we do not observe an association with other previous arbovirus infections and CZS development in the cohorts from same hospitals (7). The immunomodulatory role of rs2257167, and how this SNP influences CZS frequency, was observed especially when ZIKV infections occur in the second and third trimesters of pregnancy. This data also shows the importance of *IFNAR1* rs2257167 genetic background regulating placental gene expression culminating in CZS development. Studies using mice models and *ex vivo* placental cultures demonstrated that regions and maturity of placentas will provide different responses against ZIKV (36, 60). Generally, fetal-derived tissues developed from midgestational placenta are more restrictive to ZIKV replication (60). In fact, *in vitro* cultures show that ZIKV possesses high tropism for trophoblasts from the first trimester of pregnancy (61). Altogether, these data corroborate our findings, considering the hypothesis that ZIKV faces an IFN immunological barrier in midgestational or older placentas, while rs2257167 CG/CC carriers with higher type I and lower type III IFNs would unbalance type I/type III IFNs towards a pronounced and exacerbated type I IFN production leading to CZS susceptibility.

Notably, at term placenta from the CZS cases is associated with an increased expression of *IFIT5*, which is an important enhancer of type I IFN and a proinflammatory response (29). In parallel, placenta from CZS cases showed a decrease in *ISG15* mRNA, which was already identified as being protective from CZS ocular manifestations (62). Another role of ISG15 is to modulate IFN responses since IFNλ4 blocks type I IFN response using the ISG15 and USP18 ubiquitin system (32). Further, although *in vitro* studies strongly suggest that *TYRO3* is the main entry receptor for ZIKV (63, 64), ZIKV-infected placentas showed a decreased expression of *TYRO3*, corroborating recent findings in mice indicating that in complex organisms these receptors do not appear to be required for ZIKV infection (65). Another possibility is ZIKV downregulate entry receptors, as occurs in another viral infections (66, 67). Interestingly, here the diminished expression of *TYRO3* in CZS cases highlight its role as a signaling receptor associated with inhibition of type I IFN and general innate immune responses, as well described in other viral infections (68). This profile of augmented type I IFN associated with severity is corroborated in a ZIKV-infected mice model (31) and other TORCH infections (69, 70). However, it is noteworthy that studies in mice models demonstrated that the lack of a type I IFN response also lead to CSZ (31, 71) indicating that only optimal levels of type I IFN could possibly confer a healthy pregnancy upon Zika and probably other congenital infections.

Indeed, functionally validating our association study, a high type I IFN expression phenotypic pattern in rs2257167 CG/CC individuals was observed suggesting they cannot efficiently regulate exacerbated type I IFN, which might be one of the factors leading to CZS. Besides, the pro-inflammatory profile rs2257167 CG/CC raised placental production of inflammatory mediators upon ZIKV may contribute to an environment more susceptible to infection, as observed by Rabelo and colleagues (24).

**Figure 5:**
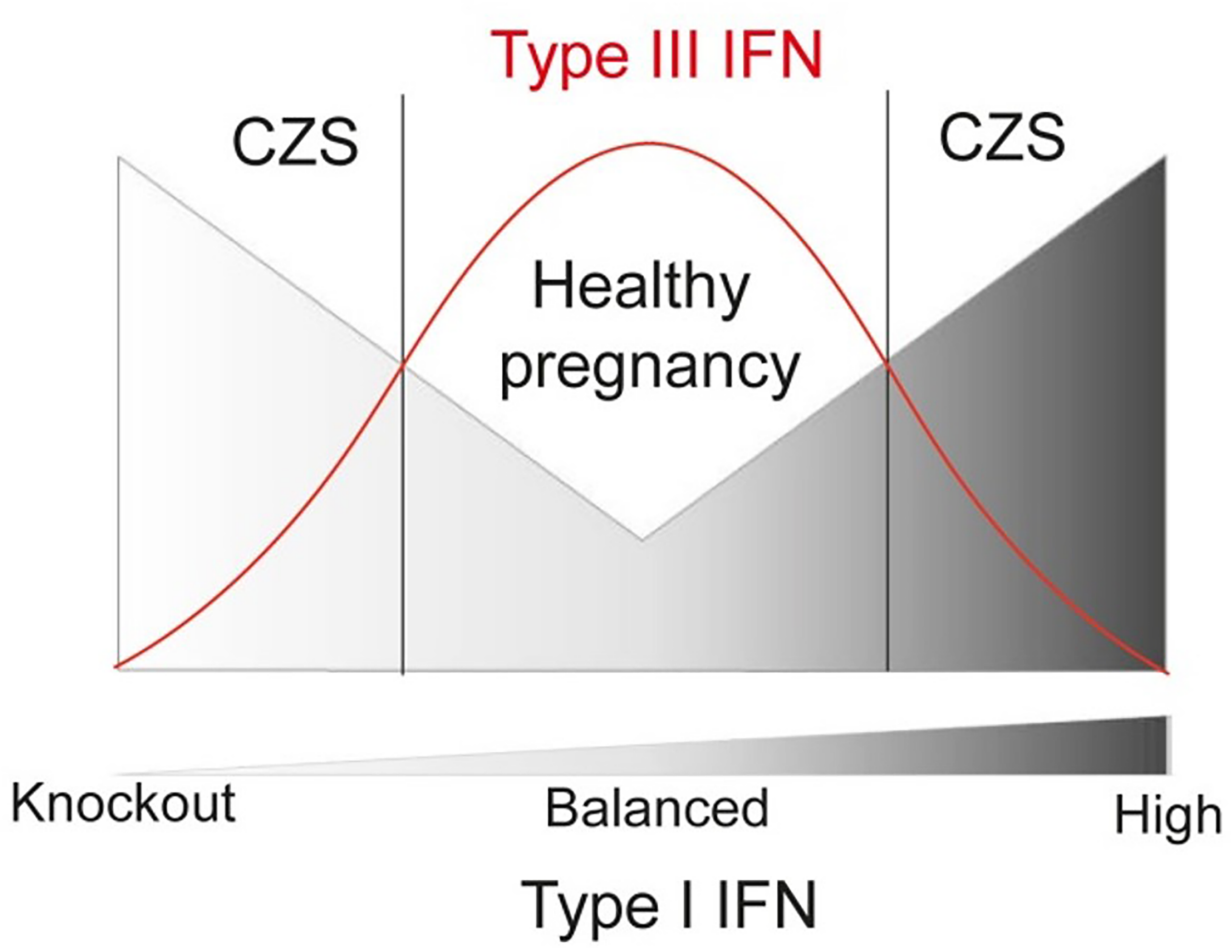
Schematic representation of functional relation between Type I and III IFN in congenital ZIKV infections regarding CZS, showing that lower risk of developing CZS is related to higher levels of IFNL and balanced levels of Type-I IFN.

Here we showed the augmented compartmentalized expression of type III interferons (*IFNL2*) during ZIKV infection. The type III IFN present a remarkable role in limiting the inflammation process with a strong antiviral activity at local level, presenting therapeutic potential (35). In this regard, exacerbated levels of type III IFNs are associated with lung barrier damage induced by SARS-CoV-2, impairing lung epithelial cell proliferation during recovery (72, 73). Altogether, the placental barrier seems to require the balance between type I and III IFN, damage and immunosuppression, to confer a healthy pregnancy upon Zika and probably other congenital infections. Hence, we can hypothesize that well-adjusted production of type I and type III IFN levels in second and third trimester of pregnancy could lead to a proper protective response to ZIKV infection in the placenta, which could prevent CZS severe outcomes (**Figure 5**).

Despite the sample size, the present association study is the bigger genetic study sampling effort of confirmed Zika cases during pregnancy (12–15). In Brazil, from April 2015 to November 2020 there are 3,590 cases of CZS with ZIKV infection confirmed by molecular diagnosis, making it difficult in obtaining large cohorts (74). Hence, genetic association of newborn *IFNAR1* rs225167 with CZS should be independently replicated in other populations. The genetic findings indicate indeed that C-allele has a prominent role in CZS risk, since different genetic models showed association in the same direction (OR-values) irrespective of the p-value. As a strategy to validate our association study, here we focused on at term placenta gene expression study as a functional validation of association study that showed characterization of placenta response to ZIKV. It is important, here we demonstrate that newborns CC/CG rs2257167 indeed present a CZS-like placenta profile. Although, the present work encourages future *in vitro* investigation of IFNs unbalance and proinflammatory responses in placenta infected by ZIKV.

**Figure 6:**
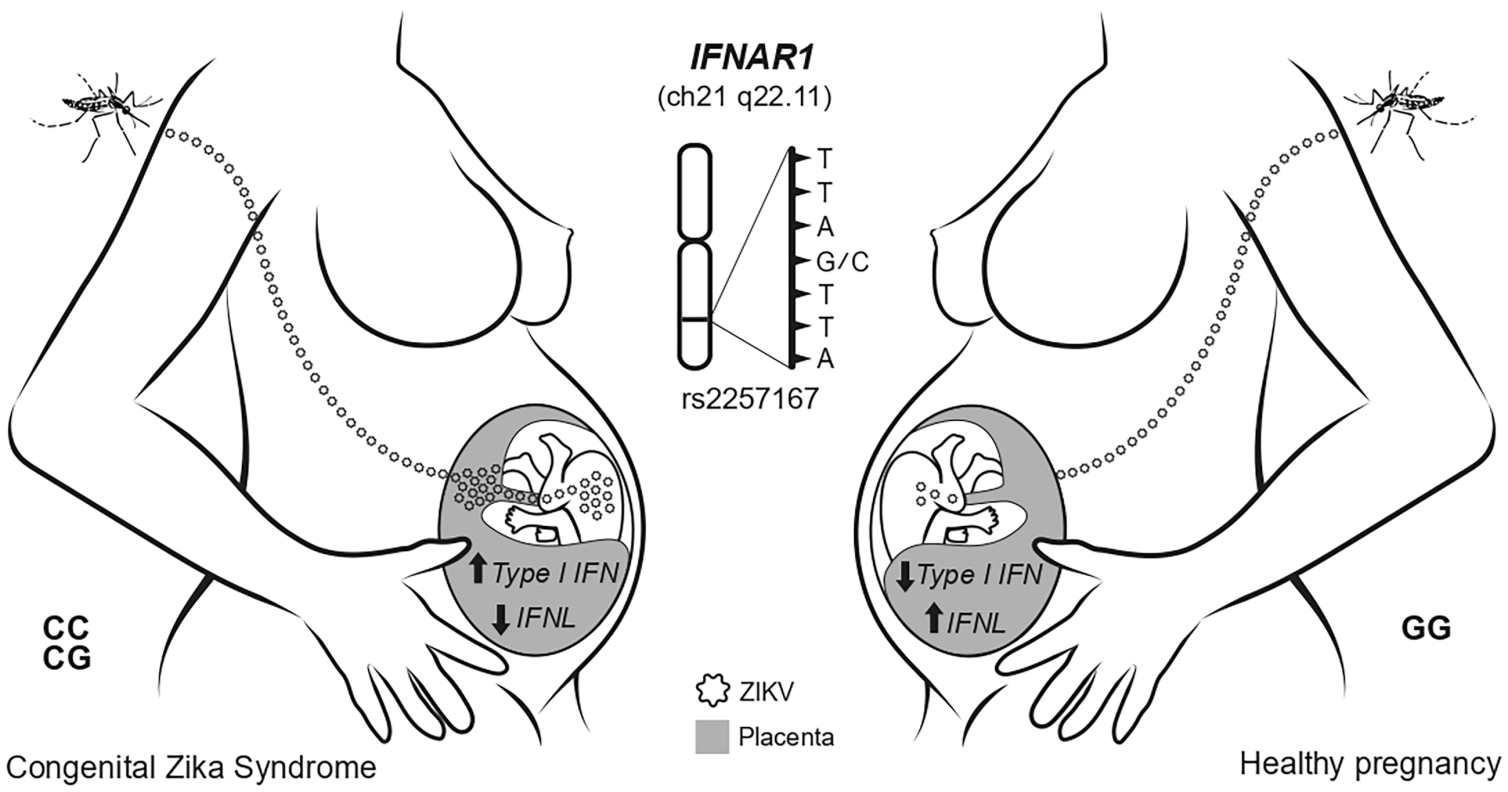
Schematic model showing the main findings of this study. The ZIKV infection during pregnancy faces placenta as an immunological barrier. In newborns with *IFNAR1* rs2257167 CG/CC genotype the high levels of type I IFN and low type III IFN in placenta, culminating with CZS.

In summary, our study showed that intensity of immune responses during ZIKV infections in humans can be regulated by *IFNAR1* rs2257167 genotypes. During pregnancy, genetic regulatory pathways control placental tissue-specific type I and type III IFN expression during ZIKV congenital infection influencing fetal neurological damage (**Figure 6**). Understanding of this novel pathway may help in the development of a custom pharmacological intervention to normalize its levels, which would likely affect and disrupt CZS development.

## Data Availability Statement

The datasets presented in this study can be found in online repositories. The names of the repository/repositories and accession number(s) can be found below: Zenodo, 10.5281/zenodo.5567327

## Ethics Statement

The studies involving human participants were reviewed and approved by the Instituto Nacional de Saúde da Mulher, da Criança e do Adolescente Fernandes Figueira, Fiocruz (IRB/CAAE: 52675616.0.000.5269). Written informed consent to participate in this study was provided by the participants’ legal guardian/next of kin.

## Author Contributions

Conceptualization: MOM, ZV, PN, and TA. Data curation: TA, DC, EP, LCG, LG, MLM, and ZV. Formal analysis: TA, FK, AS, CC, MR-A, TC, OB, FM, LF, and EC. ​Investigation: AS. Funding acquisition and resources: MM and MOM. Supervision: PN, ZV and MOM. Writing – original draft: TA. Writing – review and editing: DC, PN, ZV, and MOM​. All authors contributed to the article and approved the submitted version.

## Funding

This work was supported by the Instituto Oswaldo Cruz (Rio de Janeiro, Brazil) and by the Instituto de Tecnologia em Imunobiológicos (Rio de Janeiro, Brazil).

## Conflict of Interest

The authors declare that the research was conducted in the absence of any commercial or financial relationships that could be construed as a potential conflict of interest.

## Publisher’s Note

All claims expressed in this article are solely those of the authors and do not necessarily represent those of their affiliated organizations, or those of the publisher, the editors and the reviewers. Any product that may be evaluated in this article, or claim that may be made by its manufacturer, is not guaranteed or endorsed by the publisher.
