# Supplementary material for: Congenital Zika Syndrome Is Associated With Interferon Alfa Receptor 1
Source: Front Immunol. 2021 Nov 25;12:764746. doi: 10.3389/fimmu.2021.764746 (PMC8657619; doi:10.3389/fimmu.2021.764746)
Supplement: Supplementary file 1 [file DataSheet_1.docx]

Supplementary Material

**Supplementary Tables**

**Supplementary Table 1. Selected SNPs description**

|  |  |  |  |  | Allele frequencies (A1) | |  | | Information from annotation | |  |
| --- | --- | --- | --- | --- | --- | --- | --- | --- | --- | --- | --- |
| Chr | SNP ID | (GRCh37/hg19) | A1 | A2 | Africans | Europeans |  | | Gene | Func.refGene | Reference |
| 21 | rs2257167 | 34715699 | G | C | 0.809 | 0.850 |  | *IFNAR1* | | Missense Variant | ^39,40^ |
| 21 | rs2843710 | 34696707 | C | G | 0.650 | 0.590 |  | *IFNAR1* | | Upstream |  |
| 21 | rs2834202 | 34730954 | G | A | 0.180 | 0.268 |  | *IFNAR1* | | 3’ UTR |  |
| 21 | rs17875834 | 34721782 | C | T | 0.843 | 0.999 |  | *IFNAR1* | | Missense Variant |  |
| 19 | rs12979860 | 39738787 | C | T | 0.340 | 0.691 |  | *IFNL4* | | Intronic | ^45^ |
| 19 | rs4803222 | 39739353 | C | G | 0.251 | 0.296 |  | *IFNL4* | | 5’ UTR |  |
| 19 | rs8109886 | 39742762 | C | A | 0.148 | 0.567 |  | *IFNL4, IFNL2* | | Intergenic | ^46^ |
| 19 | rs8099917 | 39743165 | G | T | 0.036 | 0.168 |  | *IFNL4, IFNL2* | | Intergenic | ^41, 44, 46^ |
| 19 | rs368234815 | 39739155 | TT | ΔG | 0.671 | 0.377 |  | *IFNL4* | | Indel exon 1 | ^47^ |

Annotation information from ANNOVAR with references for previous association with infectious disease.

**Supplementary Table 2. Selected SNP allele descriptions**

| *IFNAR1* | | | | | |
| --- | --- | --- | --- | --- | --- |
| rs2843710 | rs2257167 | rs17875834 | rs2834202 | Haplotype frequencies | |
|  |  |  |  | Africans | Europeans |
| C | G | C | A | 0.421 | 0.581 |
| C | G | T | A | 0.245 | 0.000 |
| G | C | C | A | 0.153 | 0.125 |
| G | G | C | G | 0.109 | 0.277 |
| C | G | C | G | 0.051 | 0.000 |
| G | G | C | A | 0.019 | 0.000 |
| *IFNL2-4* | | | | | |
| rs12979860 | rs4803222 | rs8109886 | rs8099917 | Haplotype frequencies | |
|  |  |  |  | Africans | Europeans |
| T | G | A | T | 0.406 | 0.017 |
| T | C | A | T | 0.215 | 0.127 |
| C | G | A | T | 0.195 | 0.121 |
| C | G | C | T | 0.145 | 0.566 |
| T | C | A | G | 0.036 | 0.165 |

Alelle frequencies among Africans and Europeans from the 1000 Genomes Project

| Newborn (n=143) | | | | |
| --- | --- | --- | --- | --- |
|  |  | Controls no CZS (n=77) |  | Cases CZS (n=66) |
|  | Eye abnormalities  n (% of Cases) | -- |  | 27 (41%) |
|  | CNS alterations   n (% of Cases) | -- |  | 60 (91%) |
| Pregnancy trimester of first maternal Zika symptoms* | 1st | 13 (34%) |  | 26 (66%) |
|  | 2nd | 42 (75%) |  | 14(25%) |
|  | 3rd | 19 (59%) |  | 13 (41%) |
|  | Asymptomatic | 1 (7%) |  | 13 (93%) |
| Mothers (n=153) | | | | |
|  |  | Controls no CZS (n=77) |  | Cases CZS (n=76) |
|  | Eye abnormalities  n (% of Cases) | -- |  | 33 (43.4%) |
|  | CNS alterations   n (% of Cases) | -- |  | 69 (90.8%) |
| Pregnancy trimester of first maternal Zika symptoms* | 1st | 14 (30.4%) |  | 32 (69.6%) |
|  | 2nd | 42 (71.2%) |  | 17 (28.8%) |
|  | 3rd | 18 (66.7%) |  | 9 (33.3%) |
|  | Asymptomatic | 1 (5.6%) |  | 17 (94.4%) |
|  |  |  |  |  |
| Clinical information of case-control population. Newborns (n=143) and mothers (n=153) exposed to ZIKV during pregnancy, which did not develop CZS considered as controls (77 newborns and 77 mothers) and the CZS cases (66 newborns and 76 mothers). Eye abnormalities included optic nerve atrophy, chorioretinal atrophy, pigment mottling and hemorrhage, which occurs frequently and is associated with CNS alterations. CNS abnormalities include microcephaly, ventriculomegaly, cerebral calcifications, posterior fossa abnormalities, pachygyria, and lissencephaly, which were not mutually exclusive. * 2 newborns and 3 mothers with missing information of first maternal Zika symptoms. | | | | |

**Supplementary Table 3. Clinical description of cases and controls of association study**

**Supplementary Table 4. Ancestry analysis of case-control study population**

| **Newborns** | | | |
| --- | --- | --- | --- |
|  | **% African** | **% European** | **% Amerindian** |
| No CZS | 0.23 ± 0.16 | 0.61 ± 0.17 | 0.16 ± 0.11 |
| CZS | 0.30 ± 0.21 | 0.55 ± 0.20 | 0.15 ± 0.06 |
| **Mothers** | | | |
|  | **% African** | **% European** | **% Amerindian** |
| No CZS | 0.24 ± 0.17 | 0.58 ± 0.19 | 0.17 ± 0.10 |
| CZS | 0.28 ± 0.16 | 0.53 ± 0.16 | 0.19 ± 0.09 |

Average ± standard deviation percentages of African, European, or Amerindian markers, identified by genotyping 46 Ancestry Informative Markers Indels from newborns and mothers without or with CZS outcome.

**Supplementary Table 5. Association with *IFNAR1* haplotypes and CZS in newborns during ZIKV congenital infections**

|  |  |  |  |  |  | No adjustments |  | Adjusted by trimester of exposure to ZIKV and ancestry | | |
| --- | --- | --- | --- | --- | --- | --- | --- | --- | --- | --- |
|  |  |  |  | No CZS findings | CZS | OR; p-value | | |  |  |
| rs2257167 | rs2843710 | rs2834202 | rs17875834 | (%) | (%) | CI (95%) | | |  | FDR* p-value |
| G | C | A | C | 0.59 | 0.46 | ref |  | ref |  | ref |
| C | C | A | C | 0.00 | 0.03 | 7.34E+06; 0.000 |  | 5.72E+22; 0.000 |  | 0.000 |
|  |  |  |  |  |  | (7.34E+06-7.34E+06) |  | (5.72E+22-5.72E+22) |  |  |
| C | C | A | T | 0.01 | 0.02 | 0.86; 0.926 |  | 0.54; 0.73 |  | 0.852 |
|  |  |  |  |  |  | (0.04-19.70) |  | 0.02; 18.10 |  |  |
| C | G | A | C | 0.12 | 0.17 | 1.66; 0.201 |  | 0.55; 0.57 |  | 0.798 |
|  |  |  |  |  |  | (0.77-3.58) |  | 0.07-4.33 |  |  |
| G | C | G | C | 0.02 | 0.01 | 0.57; 0.627 |  | 0.26; 0.29 |  | 0.542 |
|  |  |  |  |  |  | (0.06-5.54) |  | 0.02-3.05 |  |  |
| G | G | A | C | 0.03 | 0.07 | 2.25; 0.184 |  | 0.36; 0.31 |  | 0.542 |
|  |  |  |  |  |  | (0.68-7.44) |  | 0.04-30.00 |  |  |

DNA samples from 143 newborns with congenital infection by ZIKV, which developed CZS or not, were genotyped for rs2257167, rs2843710, rs2834202, and rs17875834 SNPs at *IFNAR1*. Data were adjusted considering the trimester of the first symptoms or asymptomatic ZIKV infections and the percentage of the African and European ancestry of each individual. The total number of the genotyped samples for each SNP may vary due to genotype miscalling. Haplotype G/C (rs2257167/rs2843710) was used as haplobase. Odds ratio (OR) with 95% confidence interval (CI) and p-values. We conducted Type I error adjustment of multiple comparisons by the False Discovery Rate (FDR) method.

**Supplementary Table 6. Association study with mother *IFNAR1* and *IFNL* SNPs and CZS abnormalities**

|  | No CZS findings | |  | l CZS | |  | Adjusted by trimester of ZIKV exposure and ancestry | | | | |
| --- | --- | --- | --- | --- | --- | --- | --- | --- | --- | --- | --- |
|  | Na | % |  | Na | % |  | OR | lower | upper | p-value | FDR* p-value |
| rs2257167 | | | | | | | | | | | |
| G/G | 58 | 76.3 |  | 53 | 70.7 |  | 1 |  |  | 0.537 | 1.000 |
| C/G | 16 | 21.1 |  | 18 | 24 |  | 1.64 | 0.64 | 4.2 |  |  |
| C/C | 2 | 2.6 |  | 4 | 5.3 |  | 1.61 | 0.24 | 10.85 |  |  |
| G/G | 58 | 76.3 |  | 53 | 70.7 |  | 1 |  |  | 0.265 | 0.952 |
| C/G-C/C | 18 | 23.7 |  | 22 | 29.3 |  | 1.64 | 0.68 | 3.92 |  |  |
| G/G-C/G | 74 | 97.4 |  | 71 | 94.7 |  | 1 |  |  | 0.697 | 0.733 |
| C/C | 2 | 2.6 |  | 4 | 5.3 |  | 1.45 | 0.22 | 9.64 |  |  |
| G/G-C/C | 60 | 78.9 |  | 57 | 76 |  | 1 |  |  | 0.317 | 1.000 |
| C/G | 16 | 21.1 |  | 18 | 24 |  | 1.6 | 0.63 | 4.07 |  |  |
| Additive | 76 | 50.3 |  | 75 | 49.7 |  | 1.45 | 0.71 | 2.95 | 0.298 | 0.952 |
| rs2843710 | | | | | | | | | | | |
| G/G | 28 | 36.4 |  | 32 | 42.7 |  | 1 |  |  | 0.677 | 1 |
| C/G | 35 | 45.5 |  | 34 | 45.3 |  | 0.98 | 0.43 | 2.24 |  |  |
| C/C | 14 | 18.2 |  | 9 | 12 |  | 0.63 | 0.21 | 1.9 |  |  |
| C/C | 28 | 36.4 |  | 32 | 42.7 |  | 1 |  |  | 0.717 | 1 |
| C/G-G/G | 49 | 63.6 |  | 43 | 57.3 |  | 0.87 | 0.4 | 1.87 |  |  |
| C/C-C/G | 63 | 81.8 |  | 66 | 88 |  | 1 |  |  | 0.378 | 1 |
| G/G | 14 | 18.2 |  | 9 | 12 |  | 0.64 | 0.23 | 1.75 |  |  |
| C/C-G/G | 42 | 54.5 |  | 41 | 54.7 |  | 1 |  |  | 0.755 | 1 |
| C/G | 35 | 45.5 |  | 34 | 45.3 |  | 1.13 | 0.53 | 2.41 |  |  |
| Additive | 77 | 50.7 |  | 75 | 49.3 |  | 0.83 | 0.49 | 1.4 | 0.477 | 1 |
| rs2834202 | | | | | | | | | | | |
| A/A | 45 | 58.4 |  | 51 | 68 |  | 1 |  |  | 0.931 | 1 |
| A/G | 29 | 37.7 |  | 21 | 28 |  | 0.87 | 0.38 | 1.94 |  |  |
| G/G | 3 | 3.9 |  | 3 | 4 |  | 1.07 | 0.19 | 6.04 |  |  |
| A/A | 45 | 58.4 |  | 51 | 68 |  | 1 |  |  | 0.769 | 1 |
| A/G-G/G | 32 | 41.6 |  | 24 | 32 |  | 0.89 | 0.41 | 1.93 |  |  |
| A/A-A/G | 74 | 96.1 |  | 72 | 96 |  | 1 |  |  | 0.888 | 1 |
| G/G | 3 | 3.9 |  | 3 | 4 |  | 1.13 | 0.21 | 6.23 |  |  |
| A/A-G/G | 48 | 62.3 |  | 54 | 72 |  | 1 |  |  | 0.712 | 1 |
| A/G | 29 | 37.7 |  | 21 | 28 |  | 0.86 | 0.39 | 1.91 |  |  |
| Additive | 77 | 50.7 |  | 75 | 49.3 |  | 0.94 | 0.5 | 1.78 | 0.850 | 1 |
| rs17875834 | | | | | | | | | | | |
| C/C | 70 | 92.1 |  | 63 | 84 |  | 1 |  |  | 0.372 | 1 |
| C/T | 6 | 7.9 |  | 11 | 14.7 |  | 1.3 | 0.4 | 4.22 |  |  |
| T/T | 0 | 0 |  | 1 | 1.3 |  | 0 |  |  |  |  |
| C/C | 70 | 92.1 |  | 63 | 84 |  | 1 |  |  | 0.468 | 1 |
| C/T-T/T | 6 | 7.9 |  | 12 | 16 |  | 1.52 | 0.48 | 4.82 |  |  |
| C/C-C/T | 76 | 100 |  | 74 | 98.7 |  | 1 |  |  | 0.183 | 1 |
| T/T | 0 | 0 |  | 1 | 1.3 |  | 0 |  |  |  |  |
| C/C-T/T | 70 | 92.1 |  | 64 | 85.3 |  | 1 |  |  | 0.690 | 1 |
| C/T | 6 | 7.9 |  | 11 | 14.7 |  | 1.27 | 0.39 | 4.09 |  |  |
| Additive | 76 | 50.3 |  | 75 | 49.7 |  | 1.66 | 0.58 | 4.77 | 0.336 | 1 |
| rs12979860 | | | | | | | | | | | |
| C/C | 28 | 36.4 |  | 29 | 38.2 |  | 1 |  |  | 0.233 | 0.748 |
| C/T | 35 | 45.5 |  | 40 | 52.6 |  | 1.05 | 0.47 | 2.34 |  |  |
| T/T | 14 | 18.2 |  | 7 | 9.2 |  | 0.39 | 0.12 | 1.35 |  |  |
| C/C | 28 | 36.4 |  | 29 | 38.2 |  | 1 |  |  | 0.690 | 1 |
| C/T-T/T | 49 | 63.6 |  | 47 | 61.8 |  | 0.86 | 0.4 | 1.84 |  |  |
| C/C-C/T | 63 | 81.8 |  | 69 | 90.8 |  | 1 |  |  | 0.089 | 0.748 |
| T/T | 14 | 18.2 |  | 7 | 9.2 |  | 0.38 | 0.12 | 1.2 |  |  |
| C/C-T/T | 42 | 54.5 |  | 36 | 47.4 |  | 1 |  |  | 0.437 | 0.984 |
| C/T | 35 | 45.5 |  | 40 | 52.6 |  | 1.34 | 0.64 | 2.79 |  |  |
| Additive | 77 | 50.3 |  | 76 | 49.7 |  | 0.72 | 0.41 | 1.26 | 0.249 | 0.748 |
| rs8099917 | | | | | | | | | | | |
| T/T | 54 | 70.1 |  | 48 | 63.2 |  | 1 |  |  | 0.410 | 1 |
| G/T | 18 | 23.4 |  | 25 | 32.9 |  | 1.68 | 0.71 | 3.96 |  |  |
| G/G | 5 | 6.5 |  | 3 | 3.9 |  | 0.72 | 0.14 | 3.59 |  |  |
| T/T | 54 | 70.1 |  | 48 | 63.2 |  | 1 |  |  | 0.375 | 1 |
| G/T-G/G | 23 | 29.9 |  | 28 | 36.8 |  | 1.43 | 0.65 | 3.17 |  |  |
| T/T-G/T | 72 | 93.5 |  | 73 | 96.1 |  | 1 |  |  | 0.545 | 1 |
| G/G | 5 | 6.5 |  | 3 | 3.9 |  | 0.62 | 0.13 | 3.02 |  |  |
| T/T-G/G | 59 | 76.6 |  | 51 | 67.1 |  | 1 |  |  | 0.204 | 1 |
| G/T | 18 | 23.4 |  | 25 | 32.9 |  | 1.73 | 0.74 | 4.03 |  |  |
| Additive | 77 | 50.3 |  | 76 | 49.7 |  | 1.15 | 0.62 | 2.13 | 0.652 | 1 |
| rs8109886 | | | | | | | | | | | |
| A/A | 29 | 37.7 |  | 21 | 27.6 |  | 1 |  |  | 0.411 | 0.925 |
| C/A | 39 | 50.6 |  | 40 | 52.6 |  | 1.5 | 0.65 | 3.46 |  |  |
| C/C | 9 | 11.7 |  | 15 | 19.7 |  | 2.08 | 0.66 | 6.54 |  |  |
| A/A | 29 | 37.7 |  | 21 | 27.6 |  | 1 |  |  | 0.235 | 0.925 |
| C/A-C/C | 48 | 62.3 |  | 55 | 72.4 |  | 1.62 | 0.73 | 3.6 |  |  |
| A/A-C/A | 68 | 88.3 |  | 61 | 80.3 |  | 1 |  |  | 0.352 | 0.925 |
| C/C | 9 | 11.7 |  | 15 | 19.7 |  | 1.61 | 0.58 | 4.45 |  |  |
| A/A-C/C | 38 | 49.4 |  | 36 | 47.4 |  | 1 |  |  | 0.672 | 1 |
| C/A | 39 | 50.6 |  | 40 | 52.6 |  | 1.17 | 0.56 | 2.45 |  |  |
| Additive | 77 | 50.3 |  | 76 | 49.7 |  | 1.45 | 0.83 | 2.53 | 0.184 | 0.925 |
| rs4803222 | | | | | | | | | | | |
| G/G | 37 | 48.1 |  | 34 | 45.3 |  | 1 |  |  | 0.527 | 1 |
| C/G | 31 | 40.3 |  | 35 | 46.7 |  | 1.5 | 0.68 | 3.33 |  |  |
| C/C | 9 | 11.7 |  | 6 | 8 |  | 0.87 | 0.23 | 3.26 |  |  |
| G/G | 37 | 48.1 |  | 34 | 45.3 |  | 1 |  |  | 0.429 | 1 |
| C/G-C/C | 40 | 51.9 |  | 41 | 54.7 |  | 1.35 | 0.64 | 2.88 |  |  |
| G/G-C/G | 68 | 88.3 |  | 69 | 92 |  | 1 |  |  | 0.599 | 1 |
| C/C | 9 | 11.7 |  | 6 | 8 |  | 0.71 | 0.2 | 2.53 |  |  |
| G/G-C/C | 46 | 59.7 |  | 40 | 53.3 |  | 1 |  |  | 0.266 | 1 |
| C/G | 31 | 40.3 |  | 35 | 46.7 |  | 1.54 | 0.72 | 3.3 |  |  |
| Additive | 77 | 50.7 |  | 75 | 49.3 |  | 1.11 | 0.63 | 1.97 | 0.716 | 1 |
| rs368234815 | | | | | | | | | | | |
| TT/TT | 27 | 35.1 |  | 21 | 28.8 |  | 1 |  |  | 0.162 | 0.486 |
| TT/ΔG | 35 | 45.5 |  | 42 | 57.5 |  | 1.69 | 0.73 | 3.94 |  |  |
| ΔG/ΔG | 15 | 19.5 |  | 10 | 13.7 |  | 0.67 | 0.21 | 2.12 |  |  |
| TT/TT | 27 | 35.1 |  | 21 | 28.8 |  | 1 |  |  | 0.442 | 0.994 |
| TT/ΔG ΔG/ΔG | 50 | 64.9 |  | 52 | 71.2 |  | 1.37 | 0.61 | 3.06 |  |  |
| TT/TT- TT/ΔG | 62 | 80.5 |  | 63 | 86.3 |  | 1 |  |  | 0.145 | 0.486 |
| ΔG/ΔG | 15 | 19.5 |  | 10 | 13.7 |  | 0.47 | 0.17 | 1.31 |  |  |
| TT/TT- ΔG/ΔG | 42 | 54.5 |  | 31 | 42.5 |  | 1 |  |  | 0.075 | 0.486 |
| TT/ΔG | 35 | 45.5 |  | 42 | 57.5 |  | 1.95 | 0.93 | 4.1 |  |  |
| Additive | 77 | 51.3 |  | 73 | 48.7 |  | 0.92 | 0.53 | 1.6 | 0.78 | 1 |

DNA samples from 153 mothers with congenital infection by ZIKV with CZS or not were genotyped for *IFNAR1* SNPs rs2257167, rs2843710, rs2834202, and rs17875834; *IFNL* rs8109886, rs12979860. rs8099917, rs4803222, and rs368234815. Data were adjusted considering the trimester of the first symptoms or asymptomatic ZIKV infections and the percentage of the African and European ancestry of each individual. The total number of genotyped samples for each SNP may vary due to genotype miscalling. Major allele was used as baseline. Odds ratio (OR) with 95% confidence interval (CI) and p-values. We conducted Type I error adjustment of multiple comparisons by the False Discovery Rate (FDR) method.

**Supplementary Table 7. *IFNAR1* and *IFNL* SNP interaction in mother-child pairs and association with cephalic alteration outcomes**

|  | rs2257167 | rs2843710 | rs2834202 | rs17875834 | rs12979860 | rs8109886 | rs8099917 | rs4803222 | rs368234815 |
| --- | --- | --- | --- | --- | --- | --- | --- | --- | --- |
| logLikeRatio | 4.71 | 2.89 | 4.32 | 2.28 | 4.17 | 2.06 | 2.55 | 1.40 | 5.76 |
| p-values | 0.193 | 0.407 | 0.229 | 0.515 | 0.242 | 0.558 | 0.466 | 0.703 | 0.124 |

The DNA samples from 42 pairs of ZIKV-infected mothers and their respective newborns with or without CZS were genotyped for *IFNAR1* SNPs rs2257167, rs2843710, rs2834202, and rs17875834; *IFNL* rs8109886, rs1297986, rs8099917, and rs4803222. Estimation of Maternal, Imprinting and Interaction Effects analysis using a multinomial model to test the existence of (and estimate) the genotype relative risk parameters that increase (or decrease) the possibility that a child will be affected. No significant effects of mother or child nor mother-child genotype interaction in cephalic alteration outcomes were observed using this sample size.

**Supplementary Table 8. Demographic and clinical features of pregnant woman enrolled in placental gene expression analyses**

|  |  | Congenital ZIKV infection (n=74) | | |  | Healthy pregnancy (n=10) |
| --- | --- | --- | --- | --- | --- | --- |
|  |  | No CZS findings (n=45) |  | CZS (n=29) |  |  |
| Mothers’ age | < 40 | 33 (73.33%) |  | 26(89.66%) |  | 10 (100%) |
|  | ≥40 | 11(24.44%) |  | 0(0%) |  | 0 (0%) |
|  | Unknow | 1(2.22%) |  | 3(10.34%) |  | 0 (0%) |
|  |  |  |  |  |  |  |
| Trimester of exposure to ZIKV | 1st | 12(26.67%) |  | 18(62.07%) |  | NA |
|  | 2nd | 20(44.44%) |  | 1(3.45%) |  | NA |
|  | 3rd | 9(20.00%) |  | 0(0%) |  | NA |
|  | Asymptomatic | 1(2.22%) |  | 5(17.24%) |  | NA |
|  | Unknow | 3(6.67%) |  | 5(17.24%) |  | NA |
|  |  |  |  |  |  |  |
| ZIKV PCR in placenta | Positive | 11(24.44%) |  | 9(31.03%) |  | 0 (0%) |
|  | Negative | 34(75.56%) |  | 20(68.97%) |  | 10 (100%) |

**Supplementary Table 9. Placental gene expression according to demographic and clinical feature**

|  | Mothers’ age | | |  | | ZIKV PCR in placenta | | |  | Trimester of ZIKV infection | | | |
| --- | --- | --- | --- | --- | --- | --- | --- | --- | --- | --- | --- | --- | --- |
|  | | < 40 | ≥40 | |  | | Negative | Positive |  | 1st trimester | 2nd trimester | 3nd trimestre | Asymptomatic |
| *AIM2* | | 0.42 ± 0.15 | 0.28 ± 0.11 | |  | | 0.35 ± 0.13 | 0.52 ± 0.12 |  | 0.39 ± 0.16 | 0.39 ± 0.17 | 0.41 ± 0.16 | 0.38 ± 0.09 |
| *BCL2* | | 0.21 ± 0.07 | 0.19 ± 0.04 | |  | | 0.20 ± 0.06 | 0.23 ± 0.07 |  | 0.21 ± 0.09 | 0.20 ± 0.04 | 0.21 ± 0.04 | 0.25 ± 0.11 |
| *CARD6* | | 0.28 ± 0.13 | 0.23 ± 0.05 | |  | | 0.25 ± 0.13 | 0.29 ± 0.10 |  | 0.24 ± 0.09 | 0.26 ± 0.09 | 0.32 ± 0.09 | 0.36 ± 0.33 |
| *CARD9* | | 0.47 ± 0.23 | 0.42 ± 0.23 | |  | | 0.43 ± 0.23 | 0.56 ± 0.20 |  | 0.44 ± 0.22 | 0.49 ± 0.19 | 0.48 ± 0.16 | 0.60 ± 0.48 |
| *CCL22* | | 0.59 ± 0.39 | 0.35 ± 0.00 | |  | | 0.60 ± 0.40 | 0.54 ± 0.26 |  | 0.67 ± 0.34 | 0.46 ± 0.23 | 0.65 ± 0.36 | 0.73 ± 0.81 |
| *CCR2* | | 0.22 ± 0.08 | 0.20 ± 0.03 | |  | | 0.21 ± 0.09 | 0.23 ± 0.03 |  | 0.21 ± 0.08 | 0.21 ± 0.03 | 0.22 ± 0.04 | 0.30 ± 0.19 |
| *CCR3* | | 0.22 ± 0.05 | 0.20 ± 0.02 | |  | | 0.20 ± 0.05 | 0.24 ± 0.03 |  | 0.21 ± 0.03 | 0.21 ± 0.03 | 0.23 ± 0.05 | 0.28 ± 0.12 |
| *CCR4* | | 0.26 ± 0.10 | 0.21 ± 0.02 | |  | | 0.24 ± 0.10 | 0.27 ± 0.05 |  | 0.23 ± 0.05 | 0.25 ± 0.07 | 0.28 ± 0.07 | 0.37 ± 0.25 |
| *CCR5* | | 0.25 ± 0.11 | 0.23 ± 0.04 | |  | | 0.24 ± 0.11 | 0.26 ± 0.04 |  | 0.24 ± 0.10 | 0.24 ± 0.04 | 0.26 ± 0.05 | 0.38 ± 0.30 |
| *CD36* | | 0.31 ± 0.15 | 0.27 ± 0.07 | |  | | 0.31 ± 0.13 | 0.31 ± 0.17 |  | 0.31 ± 0.17 | 0.27 ± 0.10 | 0.33 ± 0.07 | 0.40 ± 0.28 |
| *CD74PA* | | 0.03 ± 0.02 | 0.02 ± 0.02 | |  | | 0.11 ± 0.60 | 0.03 ± 0.02 |  | 0.04 ± 0.02 | 0.03 ± 0.03 | 0.01 ± 0.01 | 0.03 ± 0.02 |
| *CIITA* | | 0.00 ± 0.00 | 0.00 ± 0.00 | |  | | 0.05 ± 0.39 | 0.00 ± 0.00 |  | 0.00 ± 0.00 | 0.00 ± 0.00 | 0.00 ± 0.00 | 0.00 ± 0.00 |
| *CLEC5A* | | 0.30 ± 0.13 | 0.26 ± 0.07 | |  | | 0.28 ± 0.11 | 0.32 ± 0.13 |  | 0.30 ± 0.14 | 0.27 ± 0.06 | 0.29 ± 0.04 | 0.39 ± 0.26 |
| *CRK* | | 0.12 ± 0.08 | 0.09 ± 0.07 | |  | | 0.13 ± 0.07 | 0.11 ± 0.09 |  | 0.14 ± 0.08 | 0.12 ± 0.08 | 0.05 ± 0.04 | 0.11 ± 0.06 |
| *DAP12* | | 0.36 ± 0.18 | 0.32 ± 0.10 | |  | | 0.35 ± 0.15 | 0.37 ± 0.20 |  | 0.36 ± 0.16 | 0.33 ± 0.12 | 0.36 ± 0.12 | 0.48 ± 0.46 |
| *DCSIGN* | | 0.31 ± 0.13 | 0.27 ± 0.07 | |  | | 0.29 ± 0.11 | 0.34 ± 0.12 |  | 0.31 ± 0.14 | 0.29 ± 0.07 | 0.31 ± 0.06 | 0.39 ± 0.24 |
| *FOXP3* | | 0.31 ± 0.18 | 0.23 ± 0.03 | |  | | 0.29 ± 0.19 | 0.32 ± 0.06 |  | 0.28 ± 0.09 | 0.26 ± 0.08 | 0.27 ± 0.04 | 0.60 ± 0.56 |
| *IDO* | | 0.42 ± 0.26 | 0.21 ± 0.06 | |  | | 0.36 ± 0.25 | 0.45 ± 0.23 |  | 0.43 ± 0.28 | 0.32 ± 0.16 | 0.39 ± 0.16 | 0.45 ± 0.49 |
| *IDO2* | | 0.30 ± 0.16 | 0.21 ± 0.05 | |  | | 0.26 ± 0.14 | 0.33 ± 0.17 |  | 0.28 ± 0.16 | 0.27 ± 0.10 | 0.29 ± 0.11 | 0.40 ± 0.34 |
| *IFI16* | | 0.40 ± 0.20 | 0.34 ± 0.10 | |  | | 0.38 ± 0.17 | 0.42 ± 0.21 |  | 0.39 ± 0.20 | 0.37 ± 0.13 | 0.43 ± 0.13 | 0.51 ± 0.41 |
| *IFI27* | | 0.32 ± 0.18 | 0.21 ± 0.06 | |  | | 0.28 ± 0.16 | 0.35 ± 0.17 |  | 0.31 ± 0.16 | 0.27 ± 0.12 | 0.32 ± 0.12 | 0.43 ± 0.39 |
| *IFI35* | | 0.35 ± 0.22 | 0.28 ± 0.08 | |  | | 0.34 ± 0.20 | 0.35 ± 0.21 |  | 0.35 ± 0.19 | 0.31 ± 0.15 | 0.33 ± 0.07 | 0.54 ± 0.56 |
| *IFI44* | | 0.42 ± 0.23 | 0.31 ± 0.07 | |  | | 0.38 ± 0.19 | 0.45 ± 0.24 |  | 0.40 ± 0.20 | 0.37 ± 0.15 | 0.45 ± 0.15 | 0.56 ± 0.54 |
| *IFI6* | | 0.31 ± 0.11 | 0.25 ± 0.07 | |  | | 0.30 ± 0.10 | 0.32 ± 0.13 |  | 0.30 ± 0.11 | 0.28 ± 0.10 | 0.31 ± 0.11 | 0.36 ± 0.22 |
| *IFIH1* | | 0.40 ± 0.23 | 0.30 ± 0.07 | |  | | 0.37 ± 0.20 | 0.40 ± 0.24 |  | 0.39 ± 0.22 | 0.33 ± 0.12 | 0.42 ± 0.12 | 0.52 ± 0.54 |
| *IFIT1* | | 0.00 ± 0.00 | 0.00 ± 0.00 | |  | | 0.05 ± 0.38 | 0.00 ± 0.00 |  | 0.00 ± 0.00 | 0.00 ± 0.00 | 0.00 ± 0.00 | 0.00 ± 0.00 |
| *IFIT2* | | 0.66 ± 0.42 | 0.46 ± 0.00 | |  | | 0.62 ± 0.40 | 0.63 ± 0.12 |  | 0.55 ± 0.17 | 0.46 ± 0.00 | 0.00 ± 0.00 | 1.14 ± 0.96 |
| *IFIT5* | | 0.18 ± 0.04 | 0.18 ± 0.02 | |  | | 0.18 ± 0.04 | 0.19 ± 0.03 |  | 0.18 ± 0.05 | 0.18 ± 0.03 | 0.19 ± 0.02 | 0.23 ± 0.07 |
| *IFNL1* | | 0.65 ± 0.39 | 0.85 ± 0.43 | |  | | 0.59 ± 0.33 | 0.85 ± 0.46 |  | 0.57 ± 0.40 | 0.71 ± 0.36 | 0.99 ± 0.45 | 0.67 ± 0.24 |
| *IFNL2* | | 1.28 ± 0.83 | 1.53 ± 0.68 | |  | | 1.17 ± 0.67 | 1.69 ± 0.95 |  | 1.18 ± 0.90 | 1.17 ± 0.64 | 1.83 ± 0.62 | 1.82 ± 1.06 |
| *IFNL3* | | 0.44 ± 0.22 | 0.57 ± 0.27 | |  | | 0.42 ± 0.19 | 0.55 ± 0.24 |  | 0.38 ± 0.15 | 0.49 ± 0.27 | 0.67 ± 0.24 | 0.48 ± 0.08 |
| *IFNL4* | | 0.54 ± 0.29 | 0.70 ± 0.36 | |  | | 0.50 ± 0.27 | 0.69 ± 0.29 |  | 0.43 ± 0.20 | 0.62 ± 0.33 | 0.85 ± 0.33 | 0.62 ± 0.21 |
| *IFNLR* | | 0.04 ± 0.17 | 0.03 ± 0.07 | |  | | 0.04 ± 0.16 | 0.00 ± 0.00 |  | 0.06 ± 0.21 | 0.02 ± 0.05 | 0.00 ± 0.00 | 0.00 ± 0.00 |
| *IFNA1* | | 0.25 ± 0.08 | 0.23 ± 0.04 | |  | | 0.24 ± 0.08 | 0.27 ± 0.03 |  | 0.24 ± 0.07 | 0.25 ± 0.05 | 0.25 ± 0.04 | 0.33 ± 0.18 |
| *IFNAR* | | 0.39 ± 0.21 | 0.27 ± 0.08 | |  | | 0.36 ± 0.18 | 0.39 ± 0.23 |  | 0.38 ± 0.22 | 0.34 ± 0.12 | 0.40 ± 0.13 | 0.46 ± 0.44 |
| *IFNB* | | 0.36 ± 0.10 | 0.35 ± 0.14 | |  | | 0.35 ± 0.10 | 0.41 ± 0.10 |  | 0.35 ± 0.10 | 0.37 ± 0.13 | 0.40 ± 0.09 | 0.35 ± 0.08 |
| *IFNG* | | 0.46 ± 0.25 | 0.31 ± 0.07 | |  | | 0.42 ± 0.24 | 0.48 ± 0.18 |  | 0.42 ± 0.12 | 0.39 ± 0.14 | 0.50 ± 0.17 | 0.60 ± 0.64 |
| *IFTM1* | | 0.31 ± 0.11 | 0.28 ± 0.06 | |  | | 0.30 ± 0.10 | 0.30 ± 0.13 |  | 0.31 ± 0.12 | 0.28 ± 0.08 | 0.34 ± 0.08 | 0.35 ± 0.18 |
| *IFTM3* | | 0.20 ± 0.04 | 0.20 ± 0.01 | |  | | 0.20 ± 0.04 | 0.20 ± 0.01 |  | 0.20 ± 0.02 | 0.20 ± 0.01 | 0.21 ± 0.01 | 0.25 ± 0.12 |
| *IL10* | | 0.33 ± 0.12 | 0.31 ± 0.10 | |  | | 0.32 ± 0.12 | 0.36 ± 0.08 |  | 0.31 ± 0.08 | 0.32 ± 0.10 | 0.35 ± 0.07 | 0.44 ± 0.29 |
| *IL12* | | 0.72 ± 0.37 | 0.46 ± 0.00 | |  | | 0.67 ± 0.36 | 0.74 ± 0.33 |  | 0.77 ± 0.37 | 0.53 ± 0.15 | 0.61 ± 0.20 | 0.91 ± 0.68 |
| *IL17* | | 0.46 ± 0.38 | 0.48 ± 0.24 | |  | | 0.47 ± 0.36 | 0.48 ± 0.25 |  | 0.34 ± 0.14 | 0.40 ± 0.28 | 0.55 ± 0.31 | 1.53 ± 0.00 |
| *IL18* | | 0.30 ± 0.11 | 0.28 ± 0.09 | |  | | 0.29 ± 0.12 | 0.32 ± 0.07 |  | 0.29 ± 0.10 | 0.30 ± 0.09 | 0.31 ± 0.07 | 0.40 ± 0.27 |
| *IL1A* | | 0.46 ± 0.26 | 0.16 ± 0.00 | |  | | 0.46 ± 0.30 | 0.38 ± 0.11 |  | 0.40 ± 0.07 | 0.32 ± 0.18 | 0.53 ± 0.00 | 0.58 ± 0.52 |
| *IL1B* | | 0.57 ± 0.25 | 0.20 ± 0.00 | |  | | 0.61 ± 0.26 | 0.48 ± 0.20 |  | 0.58 ± 0.10 | 0.41 ± 0.25 | 0.74 ± 0.00 | 0.62 ± 0.51 |
| *IL10RB* | | 0.08 ± 0.04 | 0.06 ± 0.05 | |  | | 0.15 ± 0.60 | 0.07 ± 0.04 |  | 0.09 ± 0.04 | 0.07 ± 0.05 | 0.03 ± 0.03 | 0.07 ± 0.03 |
| *IL2* | | 0.43 ± 0.16 | 0.32 ± 0.09 | |  | | 0.36 ± 0.10 | 0.51 ± 0.19 |  | 0.40 ± 0.19 | 0.43 ± 0.13 | 0.39 ± 0.13 | 0.33 ± 0.10 |
| *IL21* | | 0.45 ± 0.21 | 0.00 ± 0.00 | |  | | 0.44 ± 0.22 | 0.63 ± 0.06 |  | 0.48 ± 0.22 | 0.48 ± 0.37 | 0.00 ± 0.00 | 0.00 ± 0.00 |
| *IL22RA* | | 0.27 ± 0.11 | 0.25 ± 0.06 | |  | | 0.27 ± 0.11 | 0.29 ± 0.05 |  | 0.25 ± 0.05 | 0.27 ± 0.07 | 0.28 ± 0.05 | 0.40 ± 0.32 |
| *IL23A* | | 0.39 ± 0.19 | 0.31 ± 0.12 | |  | | 0.35 ± 0.16 | 0.45 ± 0.22 |  | 0.36 ± 0.17 | 0.39 ± 0.22 | 0.48 ± 0.20 | 0.21 ± 0.03 |
| *IL23R* | | 0.53 ± 0.28 | 0.00 ± 0.00 | |  | | 0.56 ± 0.34 | 0.47 ± 0.17 |  | 0.46 ± 0.20 | 0.48 ± 0.16 | 0.61 ± 0.03 | 0.66 ± 0.66 |
| *IL2RA* | | 0.36 ± 0.22 | 0.19 ± 0.07 | |  | | 0.32 ± 0.21 | 0.39 ± 0.19 |  | 0.30 ± 0.11 | 0.29 ± 0.14 | 0.51 ± 0.22 | 0.53 ± 0.52 |
| *IL4* | | 0.37 ± 0.19 | 0.17 ± 0.00 | |  | | 0.36 ± 0.21 | 0.32 ± 0.09 |  | 0.32 ± 0.05 | 0.26 ± 0.09 | 0.41 ± 0.00 | 0.46 ± 0.39 |
| *IL6* | | 0.37 ± 0.17 | 0.17 ± 0.07 | |  | | 0.34 ± 0.18 | 0.36 ± 0.15 |  | 0.35 ± 0.12 | 0.28 ± 0.13 | 0.36 ± 0.14 | 0.44 ± 0.42 |
| *IL7* | | 0.54 ± 0.25 | 0.30 ± 0.04 | |  | | 0.45 ± 0.26 | 0.51 ± 0.15 |  | 0.44 ± 0.17 | 0.41 ± 0.14 | 0.51 ± 0.19 | 0.87 ± 0.59 |
| *IL8* | | 0.31 ± 0.18 | 0.22 ± 0.06 | |  | | 0.28 ± 0.17 | 0.31 ± 0.15 |  | 0.29 ± 0.16 | 0.25 ± 0.12 | 0.37 ± 0.12 | 0.38 ± 0.43 |
| *IP10* | | 0.29 ± 0.12 | 0.26 ± 0.06 | |  | | 0.28 ± 0.11 | 0.32 ± 0.10 |  | 0.29 ± 0.11 | 0.28 ± 0.07 | 0.29 ± 0.05 | 0.38 ± 0.26 |
| *IRF3* | | 0.48 ± 0.26 | 0.29 ± 0.10 | |  | | 0.45 ± 0.25 | 0.47 ± 0.24 |  | 0.48 ± 0.22 | 0.38 ± 0.17 | 0.50 ± 0.23 | 0.55 ± 0.59 |
| *IRF7* | | 0.29 ± 0.09 | 0.27 ± 0.08 | |  | | 0.27 ± 0.08 | 0.32 ± 0.09 |  | 0.29 ± 0.10 | 0.28 ± 0.07 | 0.30 ± 0.05 | 0.34 ± 0.16 |
| *IRF9* | | 0.26 ± 0.09 | 0.22 ± 0.04 | |  | | 0.24 ± 0.09 | 0.27 ± 0.07 |  | 0.25 ± 0.08 | 0.24 ± 0.04 | 0.25 ± 0.04 | 0.34 ± 0.22 |
| *ISG15* | | 0.38 ± 0.27 | 0.39 ± 0.23 | |  | | 0.37 ± 0.25 | 0.40 ± 0.29 |  | 0.37 ± 0.24 | 0.36 ± 0.22 | 0.40 ± 0.14 | 0.64 ± 0.74 |
| *MARCO* | | 0.44 ± 0.28 | 0.22 ± 0.03 | |  | | 0.36 ± 0.26 | 0.50 ± 0.24 |  | 0.40 ± 0.24 | 0.31 ± 0.16 | 0.38 ± 0.18 | 1.43 ± 0.00 |
| *MCP1* | | 0.43 ± 0.22 | 0.29 ± 0.10 | |  | | 0.38 ± 0.18 | 0.47 ± 0.26 |  | 0.38 ± 0.20 | 0.41 ± 0.17 | 0.44 ± 0.17 | 0.57 ± 0.47 |
| *MIP1A* | | 0.49 ± 0.27 | 0.33 ± 0.05 | |  | | 0.43 ± 0.23 | 0.55 ± 0.31 |  | 0.47 ± 0.31 | 0.42 ± 0.14 | 0.40 ± 0.08 | 0.63 ± 0.41 |
| *MIP1B* | | 0.28 ± 0.13 | 0.23 ± 0.02 | |  | | 0.27 ± 0.13 | 0.30 ± 0.09 |  | 0.25 ± 0.07 | 0.25 ± 0.08 | 0.33 ± 0.09 | 0.45 ± 0.35 |
| *MMP2* | | 0.27 ± 0.10 | 0.24 ± 0.04 | |  | | 0.25 ± 0.08 | 0.29 ± 0.11 |  | 0.26 ± 0.09 | 0.25 ± 0.05 | 0.28 ± 0.04 | 0.35 ± 0.22 |
| *MMP9* | | 0.29 ± 0.16 | 0.19 ± 0.07 | |  | | 0.27 ± 0.15 | 0.28 ± 0.17 |  | 0.27 ± 0.13 | 0.24 ± 0.10 | 0.33 ± 0.10 | 0.36 ± 0.42 |
| *NOD2* | | 0.28 ± 0.15 | 0.17 ± 0.06 | |  | | 0.25 ± 0.12 | 0.29 ± 0.17 |  | 0.27 ± 0.15 | 0.22 ± 0.08 | 0.28 ± 0.12 | 0.35 ± 0.30 |
| *NOS* | | 0.31 ± 0.09 | 0.00 ± 0.00 | |  | | 0.30 ± 0.08 | 0.33 ± 0.08 |  | 0.36 ± 0.04 | 0.28 ± 0.12 | 0.41 ± 0.00 | 0.25 ± 0.06 |
| *NRLP1* | | 0.34 ± 0.18 | 0.23 ± 0.02 | |  | | 0.31 ± 0.17 | 0.33 ± 0.16 |  | 0.37 ± 0.22 | 0.27 ± 0.06 | 0.32 ± 0.07 | 0.41 ± 0.35 |
| *NRLP3* | | 0.43 ± 0.24 | 0.30 ± 0.03 | |  | | 0.35 ± 0.17 | 0.52 ± 0.28 |  | 0.42 ± 0.25 | 0.40 ± 0.16 | 0.45 ± 0.20 | 0.39 ± 0.38 |
| *OAS1* | | 0.42 ± 0.18 | 0.38 ± 0.16 | |  | | 0.39 ± 0.16 | 0.45 ± 0.18 |  | 0.39 ± 0.17 | 0.41 ± 0.15 | 0.43 ± 0.09 | 0.49 ± 0.43 |
| *OAS2* | | 0.37 ± 0.17 | 0.34 ± 0.16 | |  | | 0.36 ± 0.15 | 0.38 ± 0.19 |  | 0.38 ± 0.18 | 0.34 ± 0.15 | 0.51 ± 0.14 | 0.21 ± 0.13 |
| *OAS3* | | 0.35 ± 0.15 | 0.28 ± 0.04 | |  | | 0.32 ± 0.13 | 0.40 ± 0.16 |  | 0.33 ± 0.13 | 0.33 ± 0.11 | 0.39 ± 0.11 | 0.47 ± 0.37 |
| *OASL* | | 0.46 ± 0.25 | 0.37 ± 0.18 | |  | | 0.42 ± 0.23 | 0.51 ± 0.23 |  | 0.44 ± 0.23 | 0.43 ± 0.20 | 0.51 ± 0.19 | 0.58 ± 0.49 |
| *PI3K* | | 0.00 ± 0.00 | 0.08 ± 0.23 | |  | | 0.00 ± 0.00 | 0.04 ± 0.17 |  | 0.00 ± 0.00 | 0.04 ± 0.17 | 0.00 ± 0.00 | 0.00 ± 0.00 |
| *RANTES* | | 0.34 ± 0.18 | 0.22 ± 0.04 | |  | | 0.36 ± 0.20 | 0.29 ± 0.09 |  | 0.32 ± 0.03 | 0.25 ± 0.08 | 0.35 ± 0.10 | 0.47 ± 0.40 |
| *RIG1* | | 0.33 ± 0.14 | 0.27 ± 0.06 | |  | | 0.31 ± 0.11 | 0.34 ± 0.18 |  | 0.33 ± 0.16 | 0.31 ± 0.12 | 0.31 ± 0.07 | 0.38 ± 0.25 |
| *RIPK2* | | 0.37 ± 0.19 | 0.26 ± 0.06 | |  | | 0.34 ± 0.16 | 0.38 ± 0.20 |  | 0.36 ± 0.20 | 0.33 ± 0.12 | 0.37 ± 0.11 | 0.44 ± 0.36 |
| *RNASEL* | | 0.39 ± 0.24 | 0.26 ± 0.08 | |  | | 0.34 ± 0.19 | 0.43 ± 0.28 |  | 0.37 ± 0.24 | 0.33 ± 0.14 | 0.40 ± 0.16 | 0.50 ± 0.51 |
| *SOCS3* | | 0.47 ± 0.23 | 0.41 ± 0.17 | |  | | 0.46 ± 0.23 | 0.44 ± 0.18 |  | 0.44 ± 0.22 | 0.46 ± 0.16 | 0.45 ± 0.15 | 0.65 ± 0.54 |
| *SOD2* | | 0.36 ± 0.19 | 0.28 ± 0.07 | |  | | 0.34 ± 0.16 | 0.34 ± 0.21 |  | 0.36 ± 0.19 | 0.30 ± 0.13 | 0.35 ± 0.10 | 0.45 ± 0.40 |
| *STAT2* | | 0.39 ± 0.22 | 0.36 ± 0.20 | |  | | 0.37 ± 0.20 | 0.41 ± 0.25 |  | 0.37 ± 0.20 | 0.36 ± 0.20 | 0.40 ± 0.14 | 0.55 ± 0.52 |
| *STAT5* | | 0.27 ± 0.12 | 0.25 ± 0.10 | |  | | 0.27 ± 0.12 | 0.26 ± 0.11 |  | 0.25 ± 0.12 | 0.28 ± 0.12 | 0.30 ± 0.08 | 0.28 ± 0.13 |
| *STING* | | 0.45 ± 0.24 | 0.37 ± 0.17 | |  | | 0.42 ± 0.23 | 0.47 ± 0.21 |  | 0.43 ± 0.25 | 0.43 ± 0.19 | 0.45 ± 0.14 | 0.59 ± 0.52 |
| *TGFB* | | 0.29 ± 0.09 | 0.25 ± 0.06 | |  | | 0.28 ± 0.09 | 0.30 ± 0.07 |  | 0.28 ± 0.08 | 0.28 ± 0.07 | 0.31 ± 0.08 | 0.36 ± 0.23 |
| *TICAM1* | | 0.36 ± 0.26 | 0.27 ± 0.09 | |  | | 0.35 ± 0.25 | 0.34 ± 0.19 |  | 0.33 ± 0.19 | 0.28 ± 0.14 | 0.52 ± 0.18 | 0.56 ± 0.61 |
| *TLR2* | | 0.44 ± 0.23 | 0.28 ± 0.10 | |  | | 0.38 ± 0.20 | 0.48 ± 0.26 |  | 0.40 ± 0.20 | 0.39 ± 0.15 | 0.45 ± 0.17 | 0.57 ± 0.56 |
| *TLR3* | | 0.26 ± 0.08 | 0.24 ± 0.04 | |  | | 0.26 ± 0.09 | 0.26 ± 0.03 |  | 0.26 ± 0.07 | 0.25 ± 0.04 | 0.25 ± 0.03 | 0.34 ± 0.21 |
| *TLR4* | | 0.41 ± 0.24 | 0.31 ± 0.07 | |  | | 0.38 ± 0.19 | 0.44 ± 0.27 |  | 0.39 ± 0.22 | 0.35 ± 0.15 | 0.40 ± 0.12 | 0.59 ± 0.55 |
| *TLR7* | | 0.39 ± 0.27 | 0.32 ± 0.00 | |  | | 0.38 ± 0.27 | 0.40 ± 0.22 |  | 0.34 ± 0.14 | 0.28 ± 0.13 | 0.64 ± 0.11 | 0.70 ± 0.73 |
| *TLR8* | | 0.45 ± 0.24 | 0.30 ± 0.05 | |  | | 0.39 ± 0.22 | 0.52 ± 0.19 |  | 0.40 ± 0.18 | 0.41 ± 0.14 | 0.49 ± 0.20 | 0.60 ± 0.72 |
| *TNFA* | | 0.33 ± 0.19 | 0.20 ± 0.02 | |  | | 0.28 ± 0.18 | 0.35 ± 0.18 |  | 0.31 ± 0.19 | 0.27 ± 0.09 | 0.32 ± 0.12 | 0.36 ± 0.44 |
| *TNFRSF18* | | 0.61 ± 0.34 | 0.35 ± 0.00 | |  | | 0.64 ± 0.42 | 0.56 ± 0.17 |  | 0.60 ± 0.14 | 0.48 ± 0.25 | 0.60 ± 0.18 | 0.80 ± 0.73 |
| *TNFSF15* | | 0.26 ± 0.13 | 0.21 ± 0.08 | |  | | 0.24 ± 0.11 | 0.28 ± 0.14 |  | 0.25 ± 0.12 | 0.23 ± 0.08 | 0.26 ± 0.07 | 0.35 ± 0.28 |
| *TNFSF9* | | 0.57 ± 0.30 | 0.29 ± 0.00 | |  | | 0.53 ± 0.34 | 0.57 ± 0.15 |  | 0.43 ± 0.15 | 0.53 ± 0.21 | 0.66 ± 0.16 | 1.09 ± 0.80 |
| *TYRO3* | | 0.05 ± 0.07 | 0.03 ± 0.04 | |  | | 0.04 ± 0.07 | 0.04 ± 0.07 |  | 0.04 ± 0.06 | 0.05 ± 0.06 | 0.05 ± 0.10 | 0.07 ± 0.09 |
| *VIPERIN* | | 0.02 ± 0.01 | 0.01 ± 0.01 | |  | | 0.10 ± 0.61 | 0.02 ± 0.01 |  | 0.02 ± 0.01 | 0.02 ± 0.01 | 0.01 ± 0.01 | 0.02 ± 0.00 |

Mean and standard deviation of gene expression values expressed in arbitrary units normalized by housekeeping genes selected by geNorm and NormFinder as well as *18S* ribosomal RNA and *RLP13* ribosomal protein L13.

**Supplementary Figures**


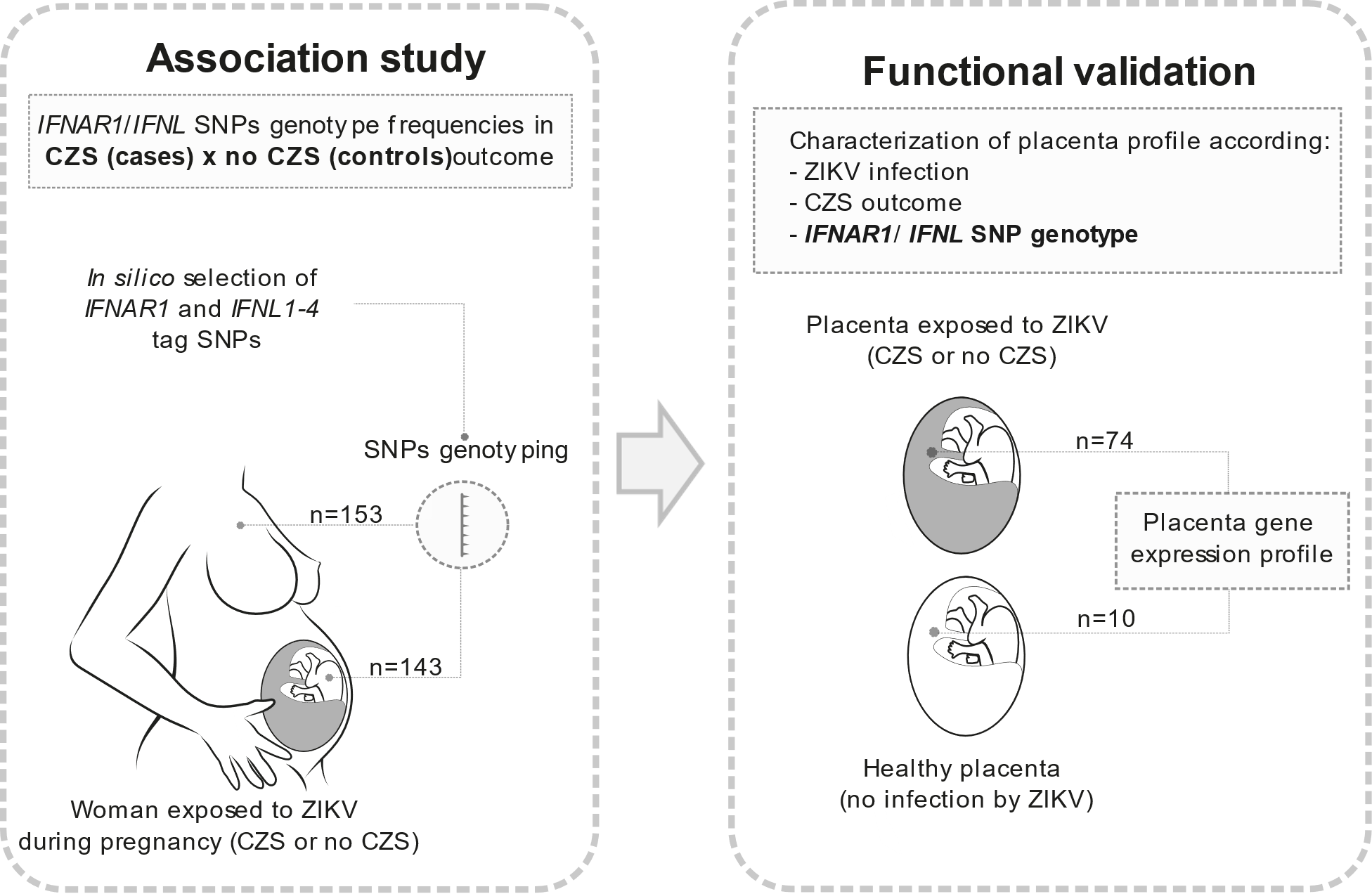


Supplementary Figure 1. Rational and workflow of sample analysis. Flowchart of Association study and functional validation of association study. For association study samples from 153 mother (77 no CZS + 76 CZS), and 143 newborns (77 no CZS + 66 CZS) exposed to ZIKV during pregnancy were genotyped for selected SNPs comprising *IFNAR1* and *IFNL* regions. *IFNAR1* and *IFNL loci* were analyzed, and SNP selected *in silico*. The SNP genotype frequencies were compared between CZS (cases) or no CZS (controls) outcome. The association study was adjusted by ancestry analysis, and the trimester of exposition to ZIKV during pregnancy. Following, for functional validation of association study, 74 placenta from pregnant woman exposed to ZIKV and 10 healthy placenta were evaluated by expression of genes related with innate responses. Gene expression utilized to characterization of placenta response according ZIKV infection, CZS outcome and *IFNAR1*/*IFNL* SNP genotypes. Values were adjusted by cofounding variables: maternal age (≥40 or <40 years old), ZIKV PCR in placenta, and the trimester of exposition to ZIKV during pregnancy.

**A B**

**
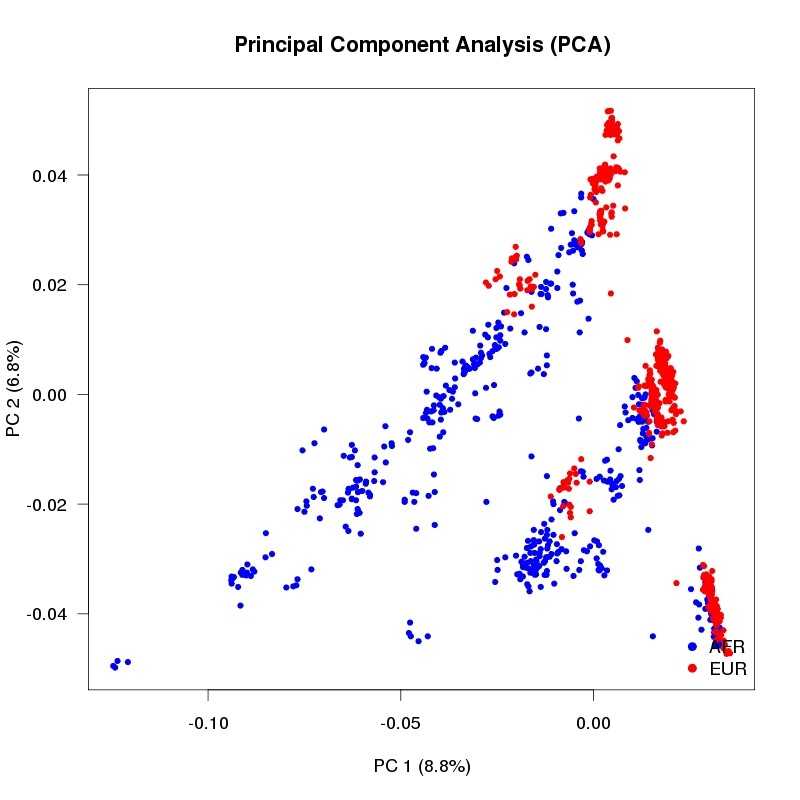

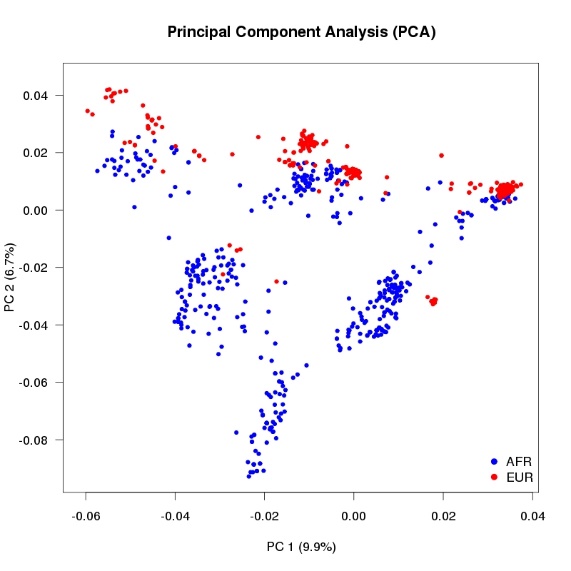
**

**C
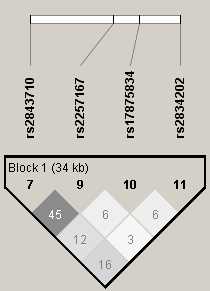
** Africans Europeans

**
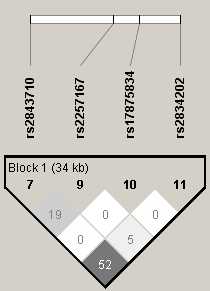
**

**D**

Africans Europeans


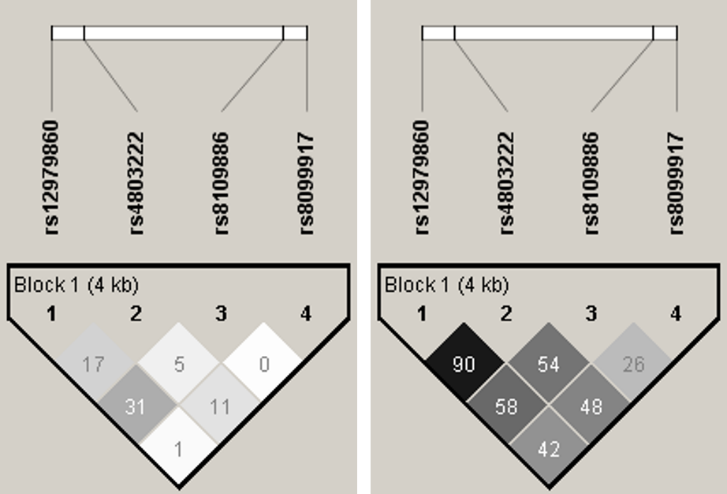


**Supplementary Figure 2.** SNP selection and linkage disequilibrium analysis. **A and B**. Principal Component Analysis (PCA) of all SNPs located in the *IFNAR1* (A) and *IFNL* (B) regions which are found among African and European populations from the 1000 Genomes Project. PC-Principal Component. **C and D.** Pattern of linkage disequilibrium (LD) for the *IFNAR1* (C) and *IFNL2-4* (D) gene regions using data from African and European populations from phase 3 of the 1000 Genomes Project. Pairwise r2 values are shown in the diamonds.


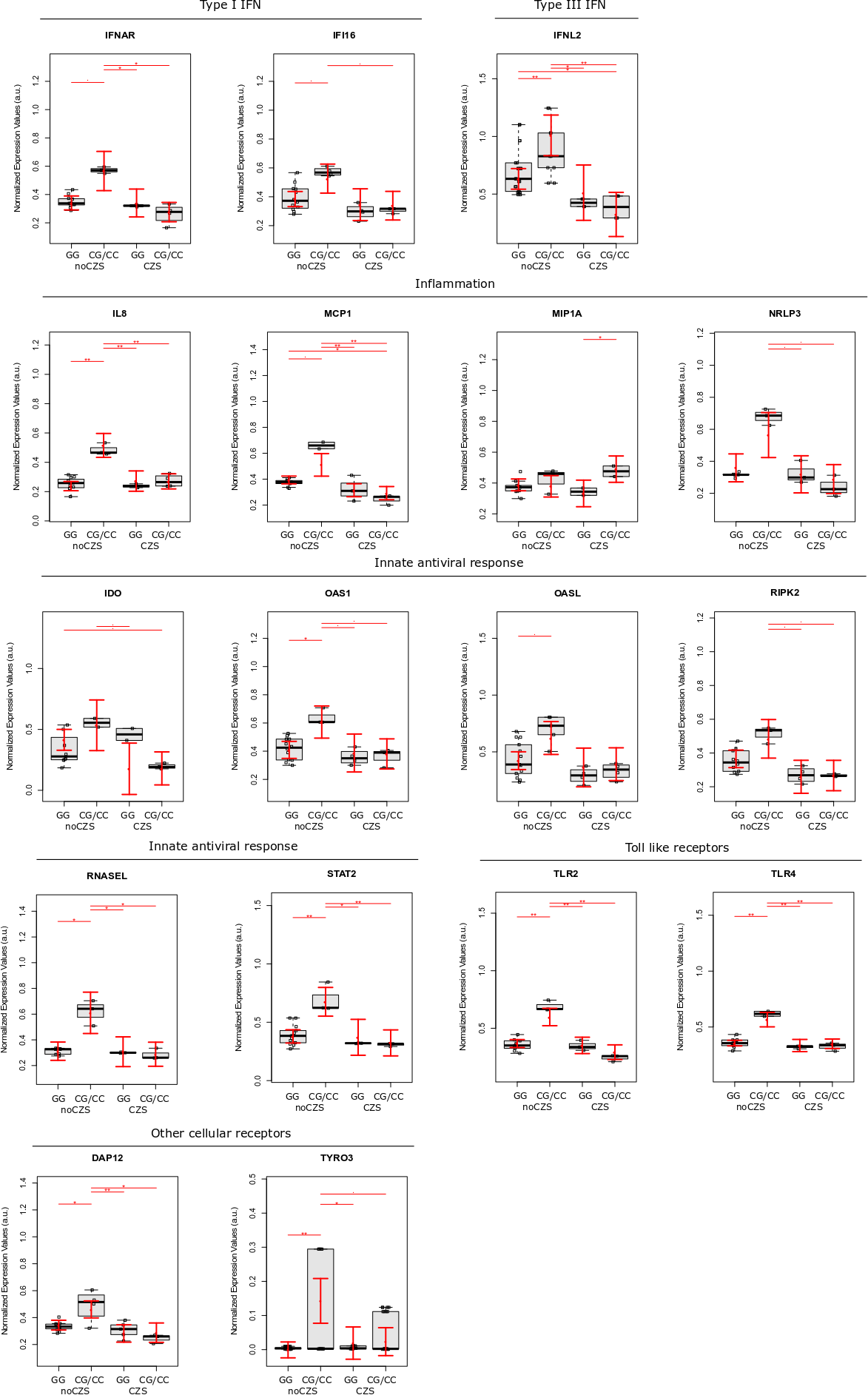


**Supplementary Figure 3**. Placental gene expression is modulated by newborn rs2257167 genotypes in ZIKV infected pregnancy. Detailed graphs of differentially expressed genes in placenta from CZS-negative cases (noCZS) clustered by rs2257167 GG (N = 14) and CG/CC (N = 10) genotypes; and placenta from CZS cases clustered by rs2257167 GG (N = 10) and CG/CC (N = 4) genotypes. Each dot corresponds to one placenta analyzed. The number of dots varies according to gene analyzed due to failed amplifications. Median and standard deviation of gene expression values are normalized by housekeeping genes selected by geNorm and NormFinder as well as *18S* ribosomal RNA and *RLP13* ribosomal protein L13 (grey boxes). Values are adjusted by mothers’ age (below or equal to/above 40 years of age) and infection trimester (trimester of pregnancy in which first Zika symptoms or asymptomatic ZIKV infections occur). P-values**≤ 0.01, * ≤ 0.05, and ≤ 0.1.
